# Functional Food Ge‐Zhi Soup Ameliorates Acute Liver Injury Through the AKT/GSK3β/PPARα Pathway

**DOI:** 10.1002/fsn3.70603

**Published:** 2025-07-21

**Authors:** Xinhua Yao, Xiaowei Lu, Duanrui Cao, Lina Huang, Zhixin Zhou, Zidong Qiu, Rui Su, Ni Zhang

**Affiliations:** ^1^ Department of Pharmacy Jiangxi University of Chinese Medicine Nanchang Jiangxi China; ^2^ State Key Laboratory for Quality Ensurance and Sustainable Use of Dao‐di Herbs, National Resource Center for Chinese Materia Medica China Academy of Chinese Medical Sciences Beijing China; ^3^ State Key Laboratory of Inorganic Synthesis and Preparative Chemistry, College of Chemistry Jilin University Changchun China

**Keywords:** acute liver injury, AKT/GSK3β/PPARα pathway, Ge‐Zhi soup, mechanism of action

## Abstract

Ge‐Zhi soup (GZS), mainly consisting of *Puerariae lobata* (Willd.) Ohwi and *Hovenia acerba* Lindl. seeds, is a traditional functional food widely consumed globally and has been proven to have considerable potential in preventing acute liver injury (ALI). However, its specific active ingredients and underlying mechanisms remain underexplored. In this study, the hepatoprotective effects, active ingredients, and underlying mechanisms of GZS were studied in ALI mice. We first determined the hepatoprotective effects of GZS and evaluated its function by analyzing biochemical parameters and histopathological changes in ALI mice. To elucidate the underlying mechanisms, an integrated strategy combining serum pharmacochemistry, network pharmacology, and non‐targeted metabolomics was employed to identify key active compounds and core targets based on the analysis of serum and tissue from the ALI mice. The results showed GZS effectively reduced the severity of liver lesions in ALI mice, revealed by histological analysis, significantly decreased the levels of AST, ALT, and MDA, and increased the levels of GSH and SOD. A total of 81 serum components were identified, including major bioactive compounds such as kaempferol, luteolin, and quercetin, as well as critical target genes such as STAT3, SRC, and PPARA. Notably, acetylcysteine was identified as a pivotal metabolite. Mechanistically, GZS's protective effects against ALI appear to be mediated through a complex regulatory network that modulates mitochondrial function and fatty acid oxidative metabolism, primarily via the AKT/GSK3β/PPARα pathway. This study elucidates the pharmacological basis and mechanisms of GZS in ALI, providing a theoretical basis for GZS as a novel functional food and therapeutic agent for ALI.

## Graphical abstract

A network among Ge‐Zhi soup–serum compounds–genes–metabolites–ALI is established. Ge‐Zhi soup regulates lipid metabolism through improving mitochondrial dysfunction. AKT/GSK3β/PPARα pathway is regulated to reduce acute liver injury.

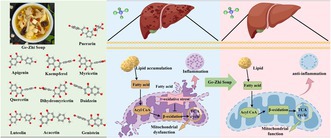

## 1 Introduction

*Puerariae lobata* (Willd.) Ohwi (PL) and *Hovenia acerba* Lindl. seeds (HA) are natural and healthy traditional food, often used in soup or tea (Zhang et al. 2023). It is widely consumed around the world. This is mainly because Ge‐Zhi soup (GZS) has a wealth of nutrients and medicinal benefits, such as alleviation of liver damage and cardiovascular diseases. GZS is composed of PL, HA, *Citri Reticulatae Pericarpium* (CR), *Chrysanthemum morifolium* Ramat. (CM), *Zizyphus jujuba* Mill (ZJ) and *Lycium barbarum* (LB). GZS has a remarkable effect on the prevention of acute liver injury (ALI) (Cao et al. 2024). Nevertheless, the mechanisms and components of GZS in its action against ALI have not been entirely elucidated. Previous studies have revealed that the core components of GZS play a role in regulating lipid metabolism. Puerarin has been found to inhibit fatty acid synthase and sterol regulatory element binding protein 1, as well as stimulate adipose triglyceride lipase (Pham et al. 2022). Therefore, we speculate that GZS may exert a function in anti‐ALI by regulating the metabolism pathway.

ALI can be triggered by various causes (Xu et al. 2020; Zou et al. 2023), including drug and alcohol misuse, exposure to toxins, hepatitis virus infection, and ischemia/reperfusion, and it may quickly lead to severe liver failure, chronic liver fat accumulation, and fibrosis (Arroyo et al. 2020). At present, the treatment for ALI primarily involves the chemical synthesis of nucleoside analogues, vitamin prevention, and liver enzyme protection drugs to manage symptoms (Chen et al. 2024; Tan et al. 2024). However, these treatments are not without limitations, exhibiting ineffectiveness and causing side effects like myopathy and acidosis. Therefore, finding new safe and effective approaches for preventing ALI and acute liver failure is essential.

The liver plays a key role in controlling the body's metabolism, including that of carbohydrates, lipids, fatty acids, proteins, and amino acids (Qian et al. 2021). ALI is characterized by cell necrosis, inflammation, and oxidative damage. Inflammation in tissues may lead to insulin resistance and increase release of free fatty acids through lipolysis, leading to lipid accumulation in the liver (Gordon and Martinez 2010). The process of hepatic lipid metabolism is managed by the intake and release of fatty acids, the synthesis of fats through lipogenesis, and the utilization of fats by β‐oxidation. When these pathways become imbalanced, liver fat starts to build up, and prolonged activation of inflammatory and fibrotic pathways can lead to more severe liver disease (Badmus et al. 2022). The studies mentioned above highlight the crucial role of metabolic balance in liver injury. Thus, focusing on metabolic homeostasis could be a promising approach for liver protection.

In this study, we try to clarify the effective substances and their possible action mechanism of GZS, which has excellent efficacy on protection of the liver. ALI mouse model was established to assess the hepatoprotective effect. An integrated strategy conjointing analysis of serum pharmacochemistry, network pharmacology and metabolomics was to characterize the active compounds of GZS and explore the potential pathway related to its synergistic effect. Computer simulation and molecular biology were used to validate and elucidate the regulatory mechanism. It was found that GZS has a significant protective effect on ALI. The network among GZS‐serum compounds‐genes‐metabolites‐ALI pinpointed key compounds, genes and metabolites, relating to the fatty acid oxidative metabolic pathway within lipid metabolism. Finally, it was confirmed that GZS has significant protective effect on ALI via modulating the AKT/GSK3β/PPARα pathway. To sum up， this research establishes a new paradigm for scientific analysis of serum compounds‐genes‐metabolite‐disease, providing a strong experimental evidence to support the further development of GZS as a novel anti‐ALI agent.

## 2 Materials and Methods

### 2.1 Samples, Chemicals and Reagents

PL stands for the dried root of *Pueraria lobata* (Willd.) Ohwi, a plant of Leguminosae. HA refers to the mature seeds of *Hovenia acerba* Lindl., a plant of the *Rhamnaceae* Juss. CR represents the dried mature pericarp of *Citrus reticulata* Blanco, a plant of the Rutaceae. CM is the dried capitulum of *Chrysanthemum morifolium* Ramat., a plant of the Asteraceae Dumort. ZJ is the dried and mature fruit of *Ziziphus jujuba* Mill., a plant of the *Rhamnaceae* Juss. LB is the dried and mature fruit of *Lycium barbarum* L., a plant of the *Solanaceae* Juss (Chinese Pharmacopoeia Commission 2020). In traditional Chinese medicine, PL and HA possess the function of detoxification, CR and CM clear the liver, and LB and ZJ can nourish theliver.

PL, HA, CR, CM, ZJ and LB were purchased from Puning Zequn Chinese medicine Co. Ltd. (Guangdon, China). All the natural food samples were identified by Prof. Fei Ge and the details of main components were presented in Figure S1 and Table S1. The bifendate tablets were purchased from Wanbangde Pharmaceutical Group Co. Ltd. (Zhejiang, China; batch No. 19J211153). Carbon tetrachloride (CAS No. C14694227, purity ≥ 98%) was purchased from Shanghai Macklin Biochemical Co. Ltd. Acetonitrile of gradient grade for liquid chromatography was purchased from EMD Millipore Corporation (Millipore, Merck). Hematoxylin–Eosin (HE) Stain Kit was obtained from Beijing Solarbio Science & Technology Co. Ltd. (Beijing, China). Aspartate aminotransferase (AST), alanine aminotransferase (ALT), malondialdehyde (MDA), glutathione (GSH), and superoxide dismutase (SOD) assay kits were from Nanjing Jiancheng Bioengineering Institute (Nanjing, China). In addition, antibodies against AKT, GSK‐3β, PPARα and GAPDH were purchased from Origene (Rockville, MD, United States). The HRP‐conjugated anti‐rabbit IgG was obtained from Proteintech (Wuhan, China). CPT1α activity assay kit was purchased from Shanghai Enzyme‐linked Biotechnology Co. Ltd. (Shanghai, China).

### 2.2 GZS Sample Preparation and Identification

According to the proportion of GZS, an accurately weighed amount of herbs was extracted twice with 500 mL water under reflux for 30 min each time. The GZS extract was concentrated under reduced pressure to 1.45 g/mL. A total of 6 reference standards, including puerarin, hesperidin, chlorogenic acid, oleanolic acid, dihydromyricetin, and *lycium barbarum* polysaccharide, were accurately weighed; then the solution was dissolved in acetonitrile to create a mixed stock solution at the appropriate concentration, which was subsequently stored at 4°C. The sample was identified by the UHPLC‐Q/TOF‐MS (Agilent, United States) equipped with a UPLC column (ZORBAX RRHD 3.0 × 100 mm, 1.8 μm).

### 2.3 Animals and Treatments

**FIGURE 1:**
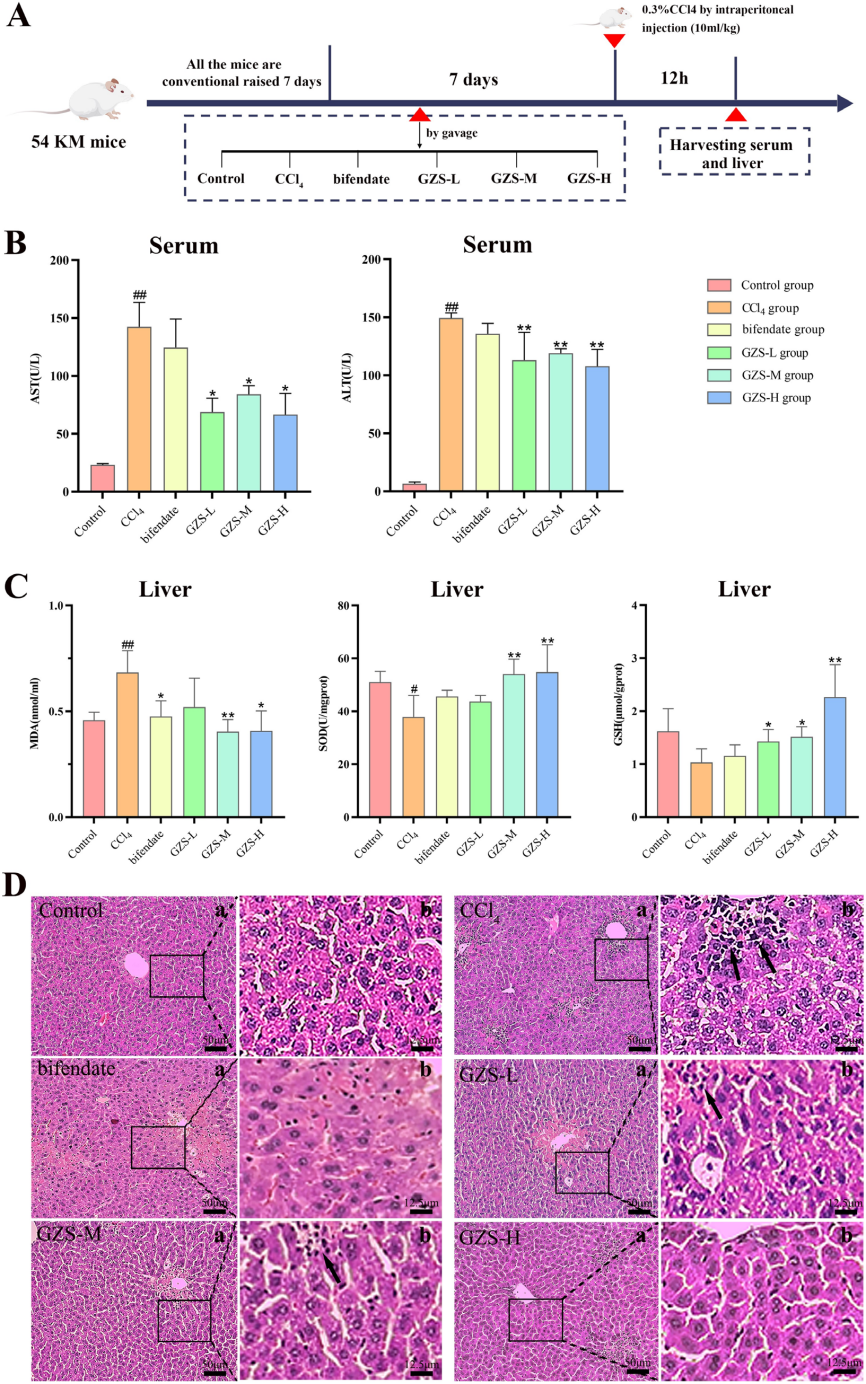
Effect of GZS intervention on ALI. (A) Process of animal grouping and treatment in pharmacodynamics research; (B) Measurement of the activities of AST and ALT in serum; (C) Detection of the level of MDA, SOD, and GSH in liver. Data are presented as the mean ± SD (*n* = 3); (D) Histopathological changes in liver tissue and effects of GZS. ^#^ *p* < 0.05, ^##^ *p* < 0.01 versus the control group; *p < 0.05, **p < 0.01 versus the CCl_4_ group ((a) magnification×50, (b) magnification×200).

SPF Kunming (KM) mice (male, weight 20–25 g, aged 6–7 weeks) were provided by the Hunan SJA Laboratory Animal Co. Ltd. (Hunan, China, SCXK (Xiang) 2021‐0004). All animal procedures were reviewed and approved by the Experimental Animal Care and Ethics Committee of the Jiangxi University of Chinese Medicine (Approval Ethics No. JZLLSC20240518). For 1 week prior to the experiment, the animals were given a standard diet and had unlimited access to food and water, while being maintained in a controlled setting (22°C ± 0.5°C, 40%–60% relative humidity, and a 12‐h light–dark cycle). Fifty‐four mice were randomly divided into six groups each containing nine mice: the control (control) group, the model (CCl_4_) group, the positive (bifendate) group (treated with 0.15 g/kg/d bifendate by gavage) (Wu et al. 2015), the treatment (GZS‐L, GZS‐M, GZS‐H) groups (treated with GZS extract 4.5, 9.0, 18 g/kg/d by gavage respectively). GZS extract and bifendate (0.15 g/kg) were administered gavage once per day for seven days. 2 h after the last administration, mice were injected with 0.3% CCl_4_ solution (i.p, 10 mL/kg, dissolved in olive oil) (Dai et al. 2023) in the CCl_4_, bifendate, and GZS groups. After 12 h, the mice underwent anesthesia, serum samples were obtained, and their livers were collected. Figure 1A provides a schematic overview of the experiment.

### 2.4 Measurement of Pathological Parameters and Fatty Acid Oxidation Related Biomarkers

According to relevant research, a few pathological parameters were investigated in the study, including the activities of AST and ALT in the serum, the level of MDA, SOD, and GSH in the liver, and histopathological assessment stained with hematoxylin–eosin. In addition, the biomarkers of fatty acid oxidation, CPT1α, were detected by enzyme‐linked immunosorbent assay (ELISA).

### 2.5 Liver Histopathological Examination

In order to observe the histopathological difference in the liver, the liver was harvested from sacrificed mice and fixed with 10% neutral buffered formaldehyde solution for 48 h. Liver tissues were prepared for histopathological examination by embedding in paraffin and staining 5 μm sections with hematoxylin and eosin.

### 2.6 Analysis of Serum Pharmacochemistry

To 500 μL of mouse serum, 1500 μL of acetonitrile was added, vortexed for 10 min, and centrifuged at 12,000 rpm for 10 min at 4°C. The supernatant was then analyzed by ultra‐high‐performance liquid chromatography coupled with time‐of‐flight mass spectrometry (UHPLC‐Q/TOF‐MS, Agilent, United States) for serum pharmacochemistry analysis. The sample was also separated on a UHPLC column (ZORBAX RRHD 3.0 × 100 mm, 1.8 μm). The mobile phase system was made up of 1% formic acid in water (A) and acetonitrile (B). The flow rate was controlled at 0.4 mL/min. The injection volume and the column temperature were preset at 3 μL and 35°C, respectively. MS data was acquired from QTOF Fusion MS equipped with a dual electrospray ionization source (Dual ESI) in positive and negative ion modes. The optimum conditions of the ion source were set as follows: spray voltage, 4.0 kV (in the positive ion mode) and −3.5 kV (in the negative ion mode); dry gas temperature, 350°C; the flow rate of dry gas, 10.0 L/min; nebulizer pressure, 35 psi. Scan mode: full mass (±); scan range: m/z 50–1000.

### 2.7 Network Pharmacology Analysis Based on Serum Pharmacochemistry

Network pharmacologic analysis was performed based on blood entry components. Serum pharmacochemistry analyses were preprocessed to identify the serum components. The targets of the serum components were predicted from the TCMSP database (https://old.tcmsp‐e.com/tcmsp.php) and the STP database (http://swisstargetprediction.ch/). The ALI‐associated targets were collected from the GeneCards database (https://www.genecards.org/) and the Online Mendelian Inheritance in Man (OMIM) database (https://omim.org/). TTD (https://db.idrblab.net/ttd/), PharmGKB (https://www.pharmgkb.org/), DrugBank (https://go.drugbank.com/), DisGeNET (https://www.disgenet.org/). We found 224 overlapping targets connecting serum components targets and ALI‐associated targets from Venny by using the bioinformatics analysis platform (http://www.bioinformatics.com.cn/). The overlapping targets were analyzed from the STRING database (https://cn.string‐db.org/cgi/input.pl) for protein–protein interaction (PPI) analysis. The disease‐component‐drug–target network and PPI network were constructed using Cytoscape 3.9.1 software. Last, KEGG enrichment analysis and Gene Ontology (GO) analysis were performed in the DAVID database (https://david.ncifcrf.gov/).

### 2.8 Analysis of Liver Tissue Metabolomics and Data Processing

100 mg liver was ground in liquid nitrogen, added to 500 μL methanol–water (4:1, *v/v*), vortexed for 5 min, and then centrifuged at 15,000 rpm for 20 min at 4°C. Mass spectrometry water was used to dilute the supernatant. Next, 100 μL methanol–water (53:47, *v/v*) was added after a vortex and centrifugation according to the same conditions, and the supernatant was analyzed via UHPLC–MS for metabolomics analysis (Want et al. 2013).

To evaluate the complete chemical variations among the control, CCl4, and GZS‐H groups, principal component analysis (PCA) was used, and partial least squares‐discriminant analysis (PLS‐DA) was utilized to discover potential biomarkers. The PCA score plot was established from the data of metabolites of each sample by using SIMCA 14.1 software. Next, the potential biomarkers were distinguished by the Kyoto Encyclopedia of Genes and Genomes (http://www.kegg.ca/). The related metabolic pathway was acquired by MetaboAnalyst 6.0.

### 2.9 Conjoint Analysis of Metabolomics and Network Pharmacology

The conjoint analysis of core genes and metabolite biomarkers was performed on the MetaboAnalyst platform (https://www.metaboanalyst.ca/) to construct the gene‐metabolite interaction network. Using Cytoscape 3.9.1, the outcome was visualized. The conjoined target genes were deemed high‐potential targets after conjoint analysis and screening. Then, the high‐potential target genes were performed to Sankey dot pathway enrichment in the bioinformatics platform (https://www.bioinformatics.com.cn/). The links between key genes and chemical components were screened for the corresponding compounds according to network pharmacology and visualized. Finally, the herbs‐compound‐target‐metabolism‐ALI network was constructed by Cytoscape 3.9.1.

### 2.10 Molecular Docking for Targets

The molecular structure of active pharmaceutical ingredients was downloaded from the PubChem database (https://pubchem.ncbi.nlm.nih.gov/#query=KZNIFHPLKGYRTM‐UHFFFAOYSA‐N). The key target proteins in the conjoint analysis of metabolomics and network pharmacology were acquired from the PDB database (https://www.rcsb.org/). The molecular docking between active pharmaceutical ingredients and key target proteins was executed by AutoDock Vina software and visualized by PyMOL software.

### 2.11 WB Analysis

The liver was lysed using RIPA lysis solution (Solarbio, China) containing 1% protease and phosphatase inhibitors, and total protein samples were collected. BCA Protein Assay Kit (Solarbio, China) was used to detect protein content. After SDS‐PAGE electrophoresis, the protein was transferred to the PVDF membrane, which was probed with primary antibodies against AKT, GSK3β, PPARα and GAPDH followed by incubation with secondary antibodies conjugated with horseradish peroxidase. Representative blots were captured using the ChemiDoc XRS+ Gel Imaging System, and the strip was analyzed with Image J software.

### 2.12 Statistical Analysis

Statistical analysis of all data was carried out by one‐way analysis of variance using SPSS 27.0 and GraphPad Prism 8.3, and values were expressed as means ± standard deviations. The least significant difference analysis was employed for groups that displayed homogeneity of variance. *p* < 0.05 was regarded as statistically significant. PLS‐DA and metabolic pathway analysis were completed using the Wekemo Bioincloud (https://www.bioincloud.tech).

## 3 Results

### 3.1 GZS Ameliorates CCl_4_ ‐Induced ALI in Mice

To evaluate the hepatoprotective effect of GZS, the measurement of pathological parameters and liver histopathological examination were performed. The process of animal grouping and treatment in pharmacodynamics research was shown in Figure 1A. Compared to the control group, the CCl_4_ group had significantly elevated serum ALT and AST levels, demonstrating successful ALI modeling (*p* < 0.01). Then, the level of AST and ALT in GZS treatment groups was significantly decreased compared with the CCl_4_ group (Figure 1B). To further verify the therapeutic effect of GZS, the levels of MDA and the activities of SOD and GSH in the liver were measured (Figure 1C). Compared with the control group, the level of MDA was significantly increased in the CCl_4_ group (*p* < 0.01). The level of MDA in the bifendate and GZS groups was significantly decreased compared with the CCl_4_ group (*p* < 0.05). In addition, the activity of SOD and GSH was decreased in the CCl_4_ group compared with the control group. The activities of SOD and GSH were significantly increased in the GZS group compared with the CCl_4_ group (*p* < 0.05). The results indicated that the GZS had apparent hepatoprotective activity.

Pathological results demonstrated that the CCl_4_ group exhibited obvious histological changes, including swollen and necrotic hepatocytes, along with infiltration of a large number of inflammatory cells (marked by arrows) into the portal area and central veins. Compared with the CCl_4_ group, pathological changes were relieved in the GZS‐treated groups (Figure 1D). The data manifested that GZS has an excellent effect on ALI.

### 3.2 Characterization and Identification of GZS Components in the Serum of ALI Mouse Model

Characterizing natural food components in serum is a fundamental technology for exploring the active substances in traditional Chinese medicine. Hence, the UHPLC‐Q/TOF‐MS was used to identify the components of serum in the GZS‐H group. The total ion chromatograms (TICs) of GZS serum components are shown in Figure S2. As a result, a total of 81 serum components were identified according to the database, as well as matching literature data and reference material verification. 12 high‐content important components from GZS metabolized in the mice serum, including recourse, name, retention time, and fragment ions, et al. were presented in Table 1, and all the serum components were shown in Table S2. Among them, 21 components were classified as the ingredients of PL; 15 components as ingredients of HA; 14 components as ingredients of CR; 12 components as ingredients of ZJ; 16 components as ingredients of CM; and 6 components as ingredients of LB.

**TABLE 1:** Details about important serum components of GZS in ALI mouse.

NO.	Resourse	Name	Formula	Calculated MS (m/z)	Measured MS (m/z)	Ion mode	Rt (min)	MS/MS fragments	References MS/MS	References
1	PL/LB/HA	Kaempferol	C_15_H_10_O_6_	285.0405	285.0423	[M − H]^−^	19.82	269.0002, 212.8675, 171.0595	269.0004, 257.0361, 240.9996	Chen et al. (2022)
2	PL	Puerarin	C_21_H_20_O_9_	417.1180	417.1173	[M + H]^+^	7.58	297.0763, 267.0615, 321.0749	267.0653, 297.0758, 321.0758	Zhang et al. (2021)
3	PL	Genistein	C_15_H_10_O_5_	269.0518	269.0513	[M − H]^−^	12.47	270.0515, 225.0339, 201.0591	240.0180, 225.0333, 201.0156	Chen et al. (2022)
4	PL	Daidzein	C_15_H_10_O_4_	255.0652	255.0654	[M + H]^+^	8.93	199.0993, 137.0243, 181.0644	137.50, 199.10	Shi et al. (2015)
5	HA	Quercetin	C_15_H_10_O_7_	301.0367	301.0392	[M − H]^−^	11.51	149.0295, 118.0255	105.0344, 149.0248	Cheng et al. (2023)
6	HA	Apigenin	C_15_H_10_O_5_	269.0455	269.0513	[M − H]^−^	12.48	239.0401, 180.0624	239.0335, 227.0335	Peng et al. (2018)
7	HA	Myricetin	C_15_H_10_O_8_	317.0303	317.0313	[M − H]^−^	27.45	317.0334, 277.0313	317.0335	Cheng et al. (2023)
8	HA	Dihydromyricetin	C_15_H_12_O_8_	319.0459	319.0521	[M − H]^−^	8.72	193.0125, 125.0256, 111.0547	125.0246, 193.0148, 301.0359	Cheng et al. (2023)
9	CM	Luteolin	C_15_H_10_O_6_	287.0550	287.0525	[M + H]^+^	19.75	213.0537, 199.0748	269.0440, 241.0493, 213.0548, 153.0183	Li, Weng, et al. (2023)
10	CM	Acacetin	C_16_H_12_O_5_	283.0612	283.0666	[M − H]^−^	14.69	268.0433, 240.0473, 195.0450	268.0376, 240.0426, 212.0476, 195.0450	Li, Yang, et al. (2023)
11	CM	Isorhamnetin	C_16_H_12_O_7_	317.0656	317.0667	[M + H]^+^	26.24	300.0190, 109.0642, 182.8915	300.0112, 287.0037, 272.0724, 151.0633	Dai and Sun (2022)
12	CM	Chrysin	C_15_H_10_O_4_	253.0506	253.0555	[M − H]^−^	13.82	176.0494, 126.9622, 77.0224	151.0199, 101.0028, 176.0433, 77.0285	Dai and Sun (2022)

Abbreviations: CM, *Chrysanthemum morifolium* Ramat; HA, *Hovenia acerba* Lindl. seeds; LB, *Lycium barbarum* L.; PL, *Puerariae lobata* (Willd.) Ohwi.

### 3.3 Network Pharmacology Analysis of GZS Serum Components and Target Genes for ALI

**FIGURE 2:**
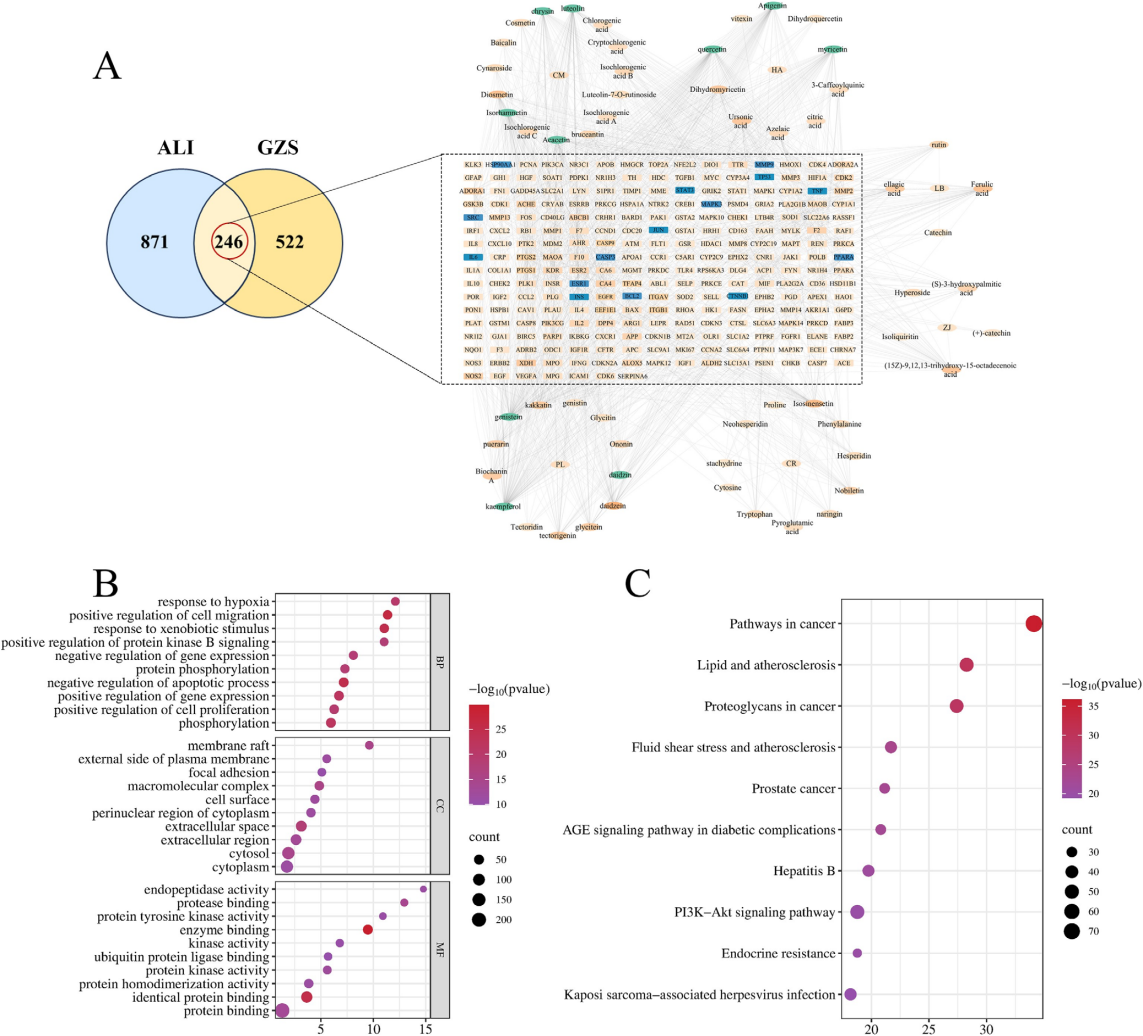
Network pharmacology analysis for GZS intervention in ALI. (A) PPI network and interaction targets of the active ingredients and ALI. (B) Results of GOBP, GOCC, and GOMF. (C) KEGG enrichment analysis.

In network analysis, we analyzed 81 serum components, and obtained 768 targets and 1117 disease‐related targets, which acted on 246 overlapping targets (Figure 2A). The ALI serum components target network included 311 nodes and 1593 edges. Among them, the top 10 targets were: kaempferol, quercetin, genistein, luteolin, apigenin, myricetin, daidzein, isorhamnetin, acacetin, and chrysin (green, Figure 2A), which were important active pharmaceutical ingredients in the treatment of ALI. A total of 246 overlapping targets were performed for PPI analysis, and 6341 pairs of PPI connections were produced (Figure S3). The top 15 genes with the highest degree values were TNF, IL6, TP53, INS, JUN, CASP3, STAT3, BCL2, CTNNB1, ESR1, SRC, MMP9, MAPK3, PPARA, HSP90AA1 (bule, Figure 2A), which were considered core genes.

### 3.4 Bioinformatics Analysis

The purpose of GO analysis was to forecast the roles of the target genes. GOBP, GOCC, and GOMF with the top 10 *p*‐values were selected and displayed using a bubble chart (Figure 2B). KEGG pathway enrichment analysis was performed to predict the signal transduction and regulated pathways. The top 10 significantly enriched pathways were shown in Figure 2C, mainly including lipid and atherosclerosis, fluid shear stress and atherosclerosis, and the PI3K‐Akt signaling pathway, which related to ALI.

### 3.5 Metabolomics Analysis

**FIGURE 3:**
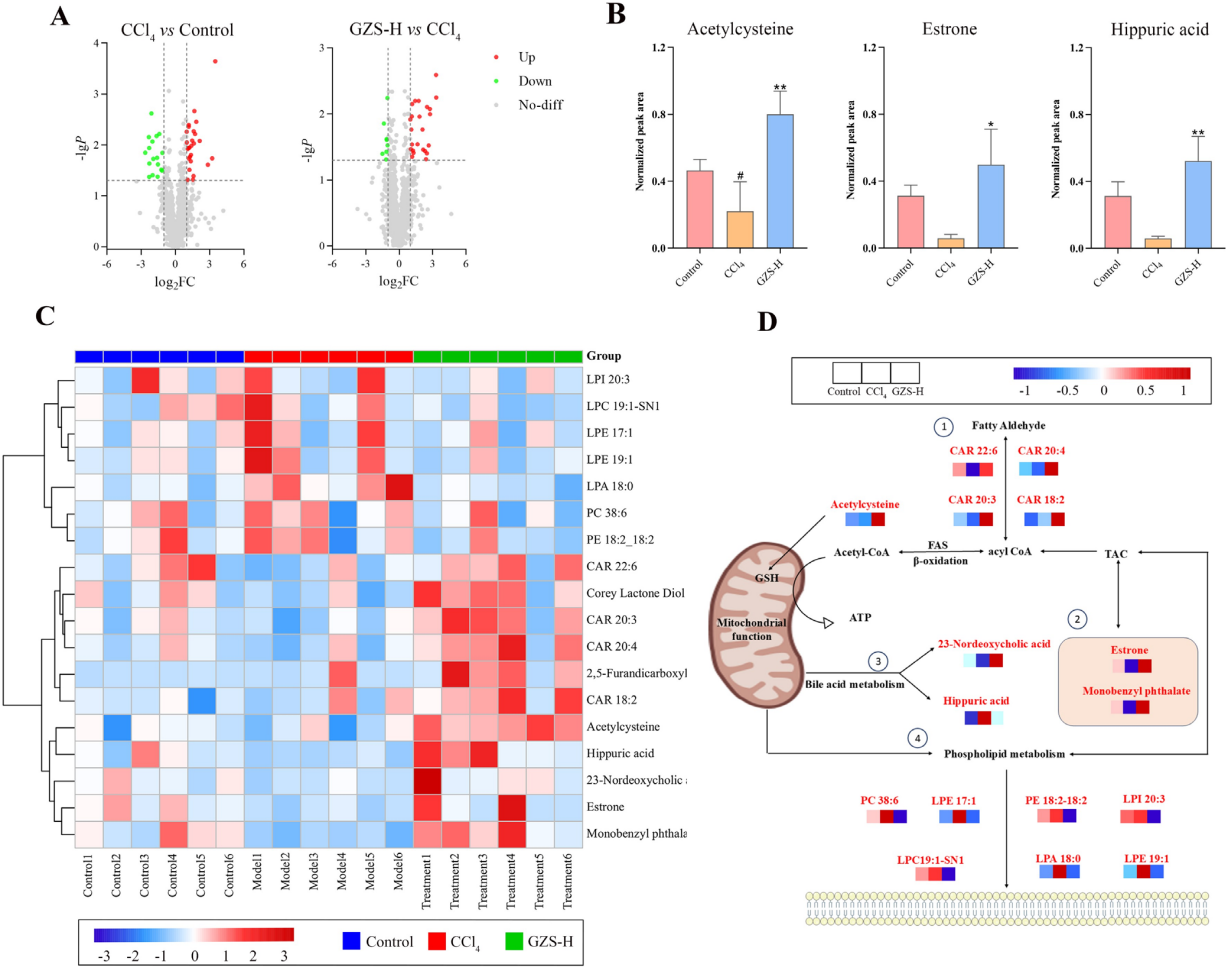
Metabolomics analysis for GZS intervention in ALI. (A) Volcano map analysis of metabolites. (B) Results of 3 biofunctionability metabolite biomarkers. (C) Clustered heatmap of the 18 metabolites related to lipid metabolism. (D) Schematic diagram of the metabolites related to lipid metabolism. ^#^ *p* < 0.05 versus the control group; *p < 0.05, **p < 0.01 versus the CCl_4_ group.

Metabolomics was performed, and the data were analyzed based on the UHPLC‐Q/TOF‐MS analysis method. A total of 1265 metabolites were identified, and the TIC spectra were shown in Figure S4. The natural distribution of different groups was exhibited by PCA. The control, CCl_4_, and GZS‐H groups were scattered and clustered clearly (Figure S5). Furthermore, the CCl_4_ group was significantly separated from the control group, and the GZS‐H group was close to the control group. To screen potential biomarkers between the groups, the metabolomics data were analyzed using a supervised method of PLS‐DA (Figure S6). In the volcano map, there were significant differences in metabolites between groups (Figure 3A). Based on VIP > 1.0 and *p*‐value < 0.05, a total of 20 metabolites were selected as potential biomarkers, which were shown in the heat map (Figure 3C) (Table S3). We analyzed the metabolite biomarkers and established associations; the result showed that metabolite biomarkers are mainly concentrated in lipid metabolism pathways (Figure 3D). The 20 metabolic biomarkers were imported into MetaboAnalyst 5.0 software. Consequently, three biofunctional metabolite biomarkers were identified, namely acetylcysteine, estrone, and hippuric acid (Figure 3B).

### 3.6 Conjoint Analysis of Metabolomics and Network Pharmacology

**FIGURE 4:**
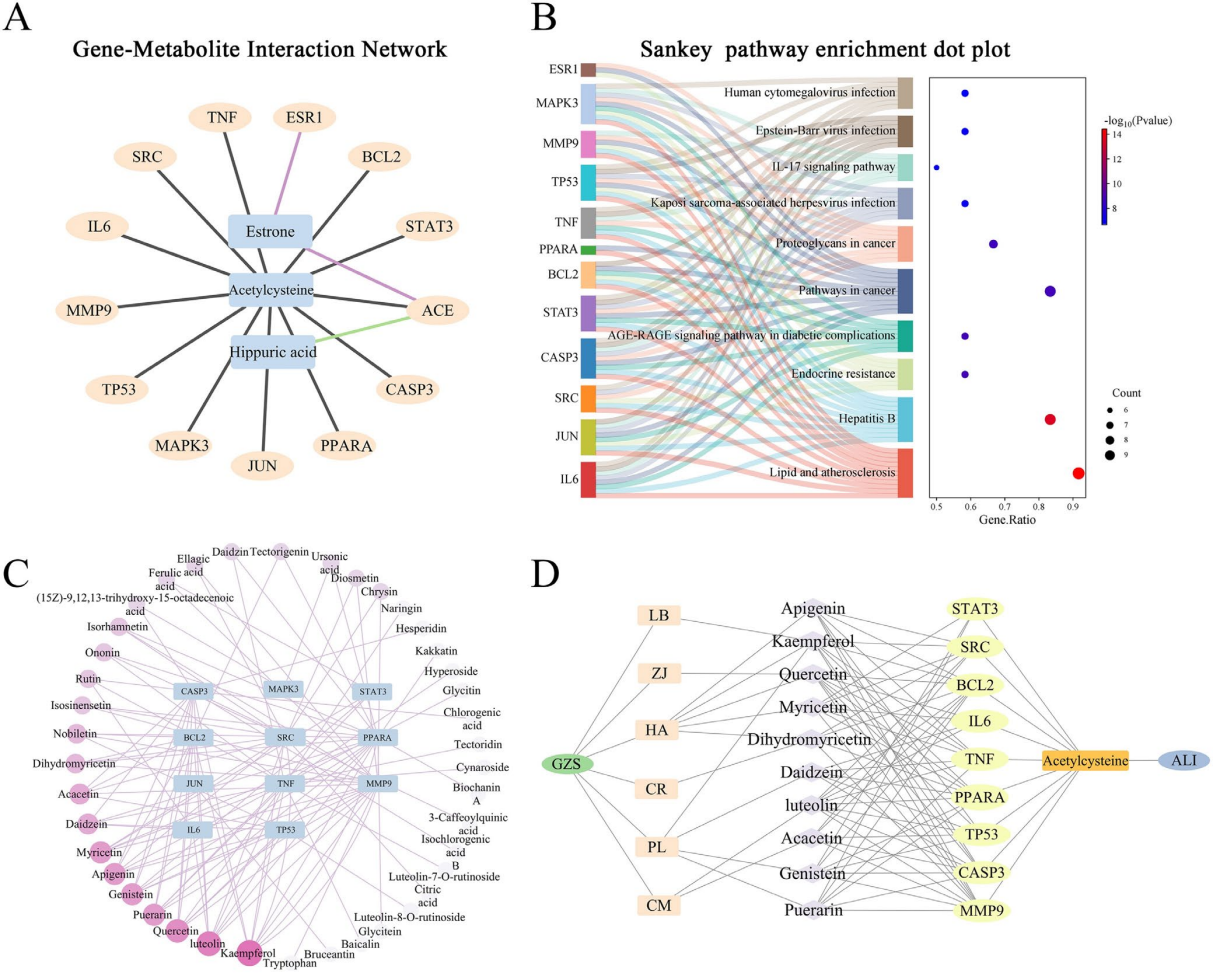
Conjoint analysis of metabolomics and network pharmacology. (A) Interaction network between genes and metabolites. (B) Pathway enrichment analysis of conjugated genes. (C) Interaction network between genes and ingredients. (D) The relationship of GZS‐serum compounds–genes–metabolites–ALI.

The conjoint analysis of core genes and metabolite biomarkers was conducted to further explore the gene‐metabolite interactions. 246 gene targets and 3 metabolites were imported into MetaboAnalyst. 63 genes associated with 3 metabolites that were analyzed, resulting in generating 66 nodes and 65 edges (Figure S7). The top 15 genes from network pharmacology analysis related to the 3 metabolites were selected, resulting in 13 key genes (TNF, IL6, TP53, JUN, CASP3, STAT3, BCL2, ESR1, SRC, MMP9, MAPK3, PPARA, ACE), with 16 nodes and 15 edges (Figure 4A). Then, a Sankey dot analysis of 12 key genes was conducted to determine the signal transduction and pathways regulated by metabolites; the results showed that the biological functions and pathways were best associated with lipid and atherosclerosis with 11 gene (Figure 4B). Moreover, a total of 81 serum compounds corresponding to 11 genes were screened, and the results showed that 40 compounds were associated with these 11 genes (Figure 4C). The top 10 corresponding compounds were: kaempferol, luteolin, quercetin, puerarin, genistein, apigenin, myricetin, daidzein, acacetin, dihydromyricetin, which also were core compounds in network pharmacology analysis as shown in Table S4. Then, 9 genes (STAT3, SRC, BCL2, IL6, TNF, PPARA, TP53, CASP3, MMP9) were associated with the 10 corresponding compounds. Finally, the natural food‐compounds‐targets‐metabolism‐ALI network was constructed as shown in Figure 4D.

### 3.7 Molecular Docking Analysis of Target Proteins

**FIGURE 5:**
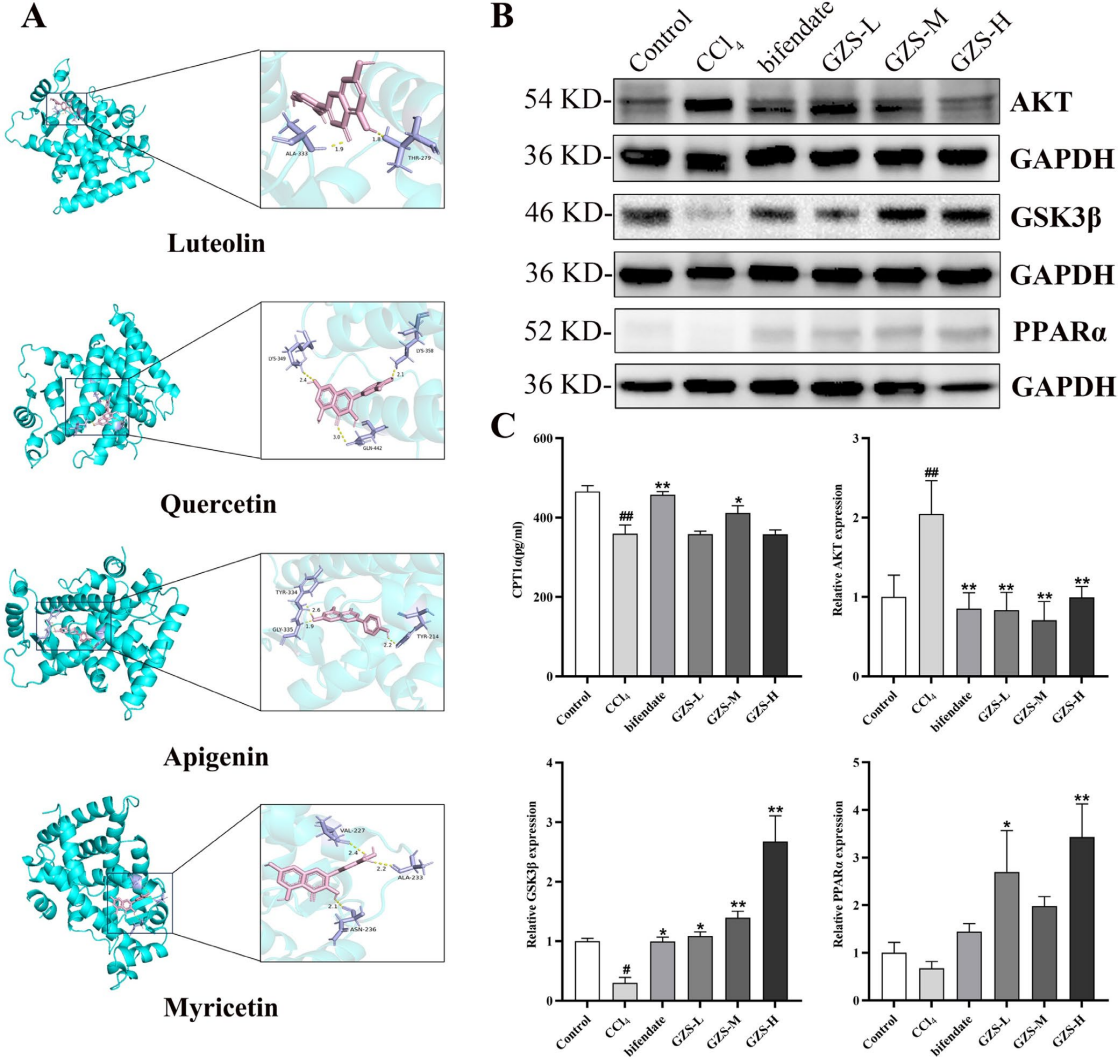
Molecular docking and protein expression associated with fatty acid oxidation. (A) Molecular docking of PPARα with luteolin, quercetin, and apigenin, myricetin. (B) AKT, GSK3β and PPARα protein expression by western blotting. (C) Protein expression results of CPT1α, AKT, GSK3β and PPARα. Data are performed as the mean ± SD (*n* = 3). ^#^ *p* < 0.05, ^##^ *p* < 0.01 versus the control group; *p < 0.05, **p < 0.01 versus the CCl_4_ group.

Finally, the 10 corresponding compounds and 9 key target proteins were analyzed and docked, and the results indicated that these compounds exhibited strong binding affinity to the core targets (Figure S8). Among them, the binding energy of PPARα was less than −7, indicating that it had strong binding ability with the active ingredient. The molecular docking of proteins and different ligands was visualized in Figure 5A. The results further verified that the GZS may act on PPARα relative pathway.

### 3.8 The Detection of Proteins Associated With Fatty Acid Oxidation

To investigate the impact of GZS on fatty acid oxidation in ALI, liver proteins linked to fatty acid oxidation were examined. The AKT, GSK3β and PPARa expressions were exhibited in Figure 5B,C. These results showed that AKT expression in the CCl_4_ group significantly increased compared with the control group (*p* < 0.01). After bifendate and GZS administration, the AKT expression was markedly decreased compared with the CCl_4_ group (*p* < 0.01). In addition, compared with the control group, the GSK3β expression was significantly decreased in the CCl_4_ group (*p* < 0.01). After bifendate and GZS treatment, the GSK3β expression was upregulated compared with the CCl_4_ group (*p* < 0.01). Then, the level of CPT1α was significantly decreased in the CCl_4_ group compared with the control group (*p* < 0.01). Compared with the CCl_4_ group, the CPT1α level of bifendate and GZS‐M groups was markedly increased (*p* < 0.05), as shown in Figure 5C. From all these results, we found that GZS alleviated liver injury by regulating fatty acid oxidation.

## 4 Discussion

The liver plays a crucial role in metabolic processes and can be easily affected by toxic substances, medications, or alcohol, leading to acute liver damage and potentially causing various metabolic disorders or even death (Xu et al. 2020). After acute liver injury, metabolic homeostasis was unbalanced, and metabolic disorders appeared, especially lipid metabolism. GZS is widely used in the diet to protect the liver, but the effective substance and mechanism are unknown. This study focused on metabolism, combining serum pharmacochemistry and network pharmacology to explore the effective substance and mechanism of GZS preventing ALI. At the animal experiment level, we demonstrated that GZS possesses a protective effect on liver injury. Combined with network pharmacological analysis, we obtained 10 important active pharmaceutical ingredients, 15 core targets, and enriched lipid and atherosclerosis pathways. We analyzed the metabolite biomarkers and established associations; the results showed that metabolite biomarkers are mainly concentrated in lipid metabolism pathways, and the metabolite biomarkers were acetylcysteine, estrone, and hippuric acid. In addition, the natural food–serum compounds–gene targets–metabolism–ALI network was constructed. In the last, our results found that GZS ameliorates ALI via the AKT/GSK3β/PPARα fatty acid oxidation pathway.

Functional foods possess intricate chemical constituents. Identifying the active constituents of functional food would facilitate the investigation of its efficacy mechanism (Zhang, Wang, et al. 2020). The integrated analysis of serum pharmacochemistry, network pharmacology, and metabolomics is a prevalent approach for screening the active constituents of functional food. Some studies have employed serum pharmacochemistry, network pharmacology, and metabolomics to explore the mechanism of Flemingia philippinensis in treating collagen‐induced arthritis rats (Qiu et al. 2025). Our investigation identified 10 principal active constituents in GZS using an integrated analysis of serum pharmacochemistry, network pharmacology, and metabolomics: kaempferol, luteolin, quercetin, puerarin, genistein, apigenin, myricetin, daidzein, acacetin, dihydromyricetin. Research indicated that kaempferol enhances oxidative stress and mitigates acute liver injury through the activation of the nuclear factor‐erythroid 2‐related factor2 (Nrf2) signaling pathway (Li, Weng, et al. 2023). Apigenin and luteolin have garnered considerable interest owing to their pronounced antioxidant, anti‐inflammatory, and immunomodulatory properties (Cai et al. 2011; Hayasaka et al. 2018). Genistein exhibits antioxidant and anti‐inflammatory effects in vitro and in vivo (Jaiswal et al. 2023; Jin et al. 2017). Importantly, genistein can improve hepatic lipid accumulation through activating the hepatic INS signaling pathway (Zhang et al. 2022). Yang et al. (2021) discovered that puerarin mitigates ALI by suppressing inflammatory responses and ZEB2 expression. Quercetin is a notable flavone within the flavonoid subgroup that has demonstrated considerable pharmacological advantages in liver disease treatment (Cao et al. 2023; Zhao et al. 2021). Myricetin, daidzein, acacetin, and dihydromyricetin have been documented to exhibit substantial protective benefits against liver injury (Chen et al. 2021; Li et al. 2021; Liou et al. 2022; Yang et al. 2021). According to lots of previous reports, the compounds screened by our method have obvious liver protective effects. The analysis of serum compound‐target‐metabolism‐disease networks highlights the multi‐dimensional and multi‐scale characteristics of functional foods in regulating complex diseases, providing a research framework from molecules to the whole for elucidating the mechanism of action of functional foods.

The liver is a key metabolic organ that regulates the metabolism of carbohydrates, proteins, and fats, with significant involvement in lipid metabolism. Luo et al. (2021) discovered that PL polysaccharides might mitigate hyperglycemia and lipid‐related metabolic dysfunction in diabetic mice. HA has been reported to influence phospholipid metabolism in alcoholic liver injury (Zhang, Yang, et al. 2020). This is consistent with our finding that GZS bioinformatics analysis of network pharmacology identified lipid and atherosclerosis signaling pathways as the first highly enriched pathways. Remarkably, all the differential metabolites in the metabolome of GZS were associated with lipid metabolism, mainly including storage lipid, structural lipids (phospholipid) and active lipids (endocrine hormone) (Figure 3D). Additionally, we discovered a biofunctional metabolic marker in metabolomics, identified as acetylcysteine, which is a finding that distinguishes our study from others. Acetylcysteine is the synthetic derivative of L‐cysteine and serves as a precursor to GSH (Raghu et al. 2021). It influences the pathophysiological mechanisms associated with oxidative stress and numerous other conditions, including mitochondrial dysfunction, apoptosis, and inflammation (Frye et al. 2019). Metabolomics findings indicated that GZS could enhance the levels of acetylcysteine (Figure 3B). Similarly, we consistently found that GZS could up‐regulate GSH in liver tissue samples. Moreover, GZS also reduced MDA content and increased SOD content in liver tissue (Figure 1C). Acetylcysteine can reduce lipid peroxidation by increasing the activities of GSH and SOD and reducing the level of MDA (Altintas et al. 2010). Interestingly, we found that GZS could affect bile acid metabolic pathways (Figure 3D). Farnesoid X receptor agonists can improve lipid disorders by regulating bile acid metabolism (Kumari et al. 2020). These data suggest that GZS can prevent ALI by improving lipid metabolism disorders.

**FIGURE 6:**
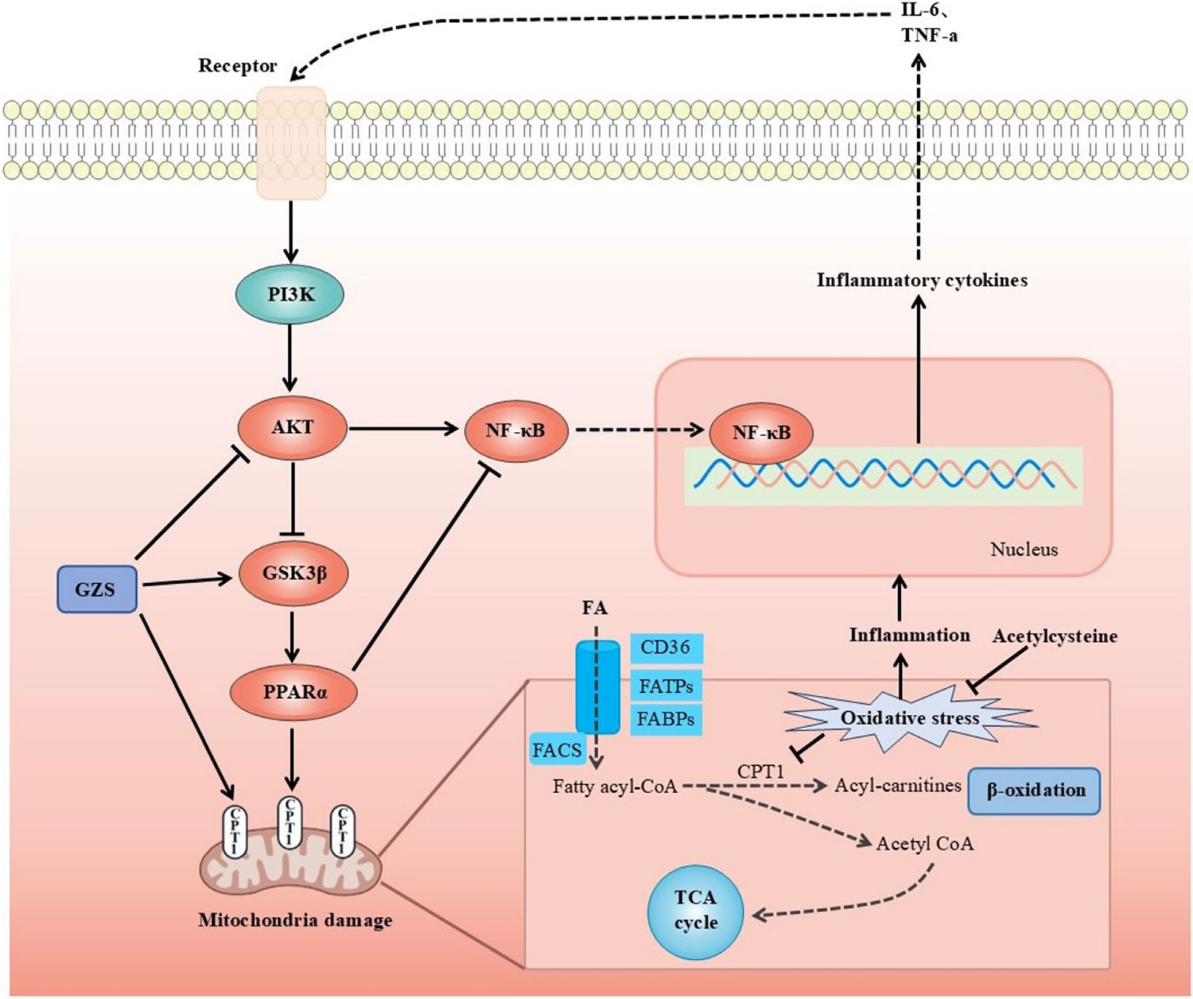
Mechanism of GZS intervention in ALI. Inflammation factors IL‐6 and TNF‐α activate AKT, which can bring about inactivation of GSK3β. GSK3β phosphorylates PPARα at serine73 in the liver. The phosphorylation of PPARα strengthens its ubiquitination, leading to increased protein turnover of the mitochondrial enzyme CPT1. The mitochondrial enzyme CPT1 is widely regarded as the most crucial enzymatic site for the regulation of fatty acid oxidation. GZS improved fatty acid oxidation via the AKT/GSK3β/PPARα pathway.

Disorders of lipid metabolism in the liver are closely related to mitochondrial dysfunction (Ma, McKeen, et al. 2020). Mitochondria serve as crucial locations for fatty acid oxidation and are a primary mechanism for energy production in catabolic animals (Amako et al. 2015). Initially, long‐chain fatty acids are activated to fatty acyl‐CoA in the cytosol and subsequently transported into the mitochondria. In the mitochondrion, fatty acyl‐CoA is processed through multiple steps to produce acetyl‐CoA, which can then be oxidized in the Krebs cycle (Ma, Wang, et al. 2020). These processes produce ATP through oxidative phosphorylation. Liver lipid homeostasis is directly regulated by mitochondrial fatty acid oxidation (Fuller et al. 2020). PPARα is a crucial regulatory element of fatty acid oxidation in the liver, and its activation can enhance inflammation, steatosis, and fibrosis in non‐alcoholic fatty liver disease (Pawlak et al. 2015). PPARα can modulate and enhance the expression of many target genes associated with diverse fatty acid metabolic pathways. It activates various enzymatic pathways associated with the uptake of fatty acids, their intracellular transport, activation, β‐oxidation, lipogenesis, ketogenesis, and the metabolism of lipoproteins and cholesterol (Tahri‐Joutey et al. 2021). Furthermore, cell factors (IL‐6, TNF‐a) activate AKT, which can promote the phosphorylation of GSK3β and bring about the inactivation of GSK3β (Tasian et al. 2014). GSK3β phosphorylates PPARα at serine73 in the liver. The phosphorylation of PPARα strengthens its ubiquitination, leading to increased protein turnover and decreased activity (Hinds et al. 2016). The mitochondrial enzyme CPT1 is widely regarded as the most crucial enzymatic site for the regulation of fatty acid oxidation (Figure 6). Research indicates that the regulation of the AKT/GSK3β pathway mitigates acetaminophen‐induced liver damage (Li et al. 2018; Fan et al. 2018). Zuniga et al. (2011) discovered that dietary supplementation with n‐3 polyunsaturated fatty acids could activate PPARα and ameliorate ischemia–reperfusion (I/R) liver injury in rats. However, there have been no studies manifesting that acute liver damage could be attenuated via the AKT/GSK3β/PPARα pathway. In our study, molecular docking was conducted between 10 corresponding compounds and 9 key target proteins; among these targets, PPARα had a strong binding ability with the active ingredient (Figure 5A). In addition, the AKT expression was elevated, but the levels of GSK3β and CPT1α were decreased in ALI, further suggesting that mitochondrial fatty acid oxidation plays a role in the development of ALI. After GZS administration, the proteins associated with fatty acid oxidation were restored in the liver (Figure 5B,C). This confirmed that GZS could protect ALI by promoting fatty acid oxidation. In this study, we first proposed that the regulation of the AKT/GSK3β/PPARα fatty acid oxidation pathway was useful for reducing liver damage. This axis may be a novel strategy against ALI.

There are several limitations to this study. First, the components entering the mouse serum are not all the components in the GZS. They are the metabolites of GZS absorbed through the gastrointestinal tract. There are still many active secondary metabolites in the serum that have not been identified. To address this, we compared fragment ions of the compounds in the serum with the literature studies and tried our best to identify 81 blood‐entering components of GZS. Next, although we speculated that mitochondrial dysfunction would affect the normal metabolism of lipids, we did not observe the mitochondrial morphology or detect various proteins related to mitochondrial function in the liver of mice. Nevertheless, we detected the content of CPT1 in the liver, which can partially represent the function of liver mitochondria. While this study identified 10 primary active components in GZS, further investigation is required at the cellular level to substantiate their effects on liver injury.

## 5 Conclusion

In summary, this study establishes a new paradigm for researching the effective substances and mechanisms of action of natural food, using GZS as a case study. This includes mass spectrometry characterization of the material basis, network pharmacology, metabolomic system analysis, and Western blot experimental validation. A comprehensive investigation on ALI model mice revealed that GZS effectively reduced serum AST and ALT concentrations, elevated hepatic MDA levels, and decreased hepatic GSH and SOD levels. Additionally, GZS ameliorated the histopathological changes associated with ALI. High‐resolution mass spectrometry identified 81 serum components in ALI‐affected mice. By employing an integrated approach of serum medicinal chemistry, network pharmacology, and untargeted metabolomics, we identified potential key pharmacodynamic substances such as kaempferol, quercetin, genistein, luteolin, apigenin, myricetin, daidzein, isorhamnetin, acacetin, and chrysin, along with key genes including STAT3, SRC, BCL2, IL6, TNF, PPARA, TP53, CASP3, and MMP9. Acetylcysteine was identified as a key metabolite. Mechanistically, comprehensive molecular biology validation suggests that the anti‐ALI effect of GZS may be mediated through an integrated regulatory network involving these key compounds, genes, and metabolites, primarily targeting mitochondrial function within lipid metabolism and fatty acid oxidative pathways, potentially regulated via the AKT/GSK3β/PPARα pathway. This research presents a robust methodology for investigating the efficacy of substances and understanding the mechanisms of action in natural food, thereby establishing a new framework for employing an integrated strategy to elucidate the scientific effect of natural food.

## Author Contributions

**Xinhua Yao:** investigation (lead), methodology (equal), visualization (lead), writing – original draft (lead). **Xiaowei Lu:** visualization (equal), writing – original draft (equal). **Duanrui Cao:** investigation (equal). **Lina Huang:** investigation (equal). **Zhixin Zhou:** investigation (equal). **Zidong Qiu:** conceptualization (equal), supervision (equal), writing – review and editing (equal). **Rui Su:** conceptualization (equal), supervision (equal), writing – review and editing (equal). **Ni Zhang:** conceptualization (lead), funding acquisition (lead), project administration (equal), writing – review and editing (equal).

## Conflicts of Interest

The authors declare no conflicts of interest.

## Supporting information


**Table S1:** Details about 6 key components of GZS.
**Table S2:** Details about 81 serum components of GZS in ALI mouse.
**Table S3:** Differential metabolites.
**Table S4:** Metabolomics combined with network pharmacology ranking.
**Figure S1:** Identification of natural food key components.
**Figure S2:** Total Ion Chromatogram of serum components.
**Figure S3:** PPI.
**Figure S4:** Total Ion Chromatogram of metabolites.
**Figure S5:** PCA score plot.
**Figure S6:** OPLS‐DA model.
**Figure S7:** Associated analysis of genes and metabolites.
**Figure S8:** Molecular docking of 10 corresponding compounds and 9 key target proteins.

## Data Availability

The authors confirm that the data supporting the findings of this study are available within the article or its Supporting Information—S1.
