# Supplementary material for: Functional Food Ge‐Zhi Soup Ameliorates Acute Liver Injury Through the AKT/GSK3β/PPARα Pathway
Source: Food Sci Nutr. 2025 Jul 21;13(7):e70603. doi: 10.1002/fsn3.70603 (PMC12280231; doi:10.1002/fsn3.70603)
Supplement: Supplementary file 1 — Table S1: Details about 6 key components of GZS. Table S2: Details about 81 serum components of GZS in ALI mouse. Table S3: Differential metabolites. Table S4: Metabolomics combined with network pharmacology ranking. Figure S1: Identification of natural food key components. Figure S2: Total Ion Chromatogram of serum components. Figure S3: PPI. Figure S4: Total Ion Chromatogram of metabolites. Figure S5: PCA score plot. Figure S6: OPLS‐DA model. Figure S7: Associated analysis of genes and metabolites. Figure S8: Molecular docking of 10 corresponding compounds and 9 key target proteins. [file FSN3-13-e70603-s001.docx]

**Supplementary Material**

**Tab. S1** Details about 6 key components of GZS.

**Tab. S2** Details about 81 serum components of GZS in ALI mouse.

**Tab. S3** Differential metabolites.

**Tab. S4** Metabolomics combined with network pharmacology ranking.

**Fig. S1** Identification of natural food key components.

**Fig. S2** Total Ion Chromatogram of serum components.

**Fig. S3** PPI.

**Fig. S4** Total Ion Chromatogram of metabolites.

**Fig. S5** PCA score plot.

**Fig. S6** OPLS-DA model.

**Fig. S7** Associated analysis of genes and metabolites.

**Fig. S8** Molecular docking of 10 corresponding compounds and 9 key target proteins.

| **Table S1 Details about 6 key components of GZS.** | | | | | | | | | | |  |
| --- | --- | --- | --- | --- | --- | --- | --- | --- | --- | --- | --- |
| **NO.** | **Resourse** | **Name** | **Formula** | **Calculated MS**  **(m/z)** | **Measured MS**  **(m/z)** | **Ion Mode** | **Rt(min)** | **MS/MS Fragments** | **References MS/MS** | **References** | |
| 1 | PL | Puerarin | C_21_H_20_O_9_ | 417.1180 | 417.1178 | [M+H]^+^ | 7.71 | 297.0755, 267.0649, 268.0714 | 267.0653, 297.0758, 321.0758 | (Zhang et al., 2021) | |
| 2 | CR | Hesperidin | C_28_H_34_O_15_ | 611.1970 | 611.1851 | [M+H]^+^ | 11.47 | 303.0805, 153.0150, 195.0248 | 431.1379, 413.1202, 395.1131, 369.0974, 315.0864, 303.0857, 263.0544, 219.0281, 195.0285, | (Luo et al., 2023) | |
| 3 | HA/CR | Dihydromyricetin | C_15_H_12_O_8_ | 319.0459 | 319.0529 | [M-H]^-^ | 8.68 | 193.0169, 125.0269, 137.0256 | 125.0246, 193.0148, 301.0359 | (Cheng, Sun, Hu, Deng, Zhang & Li, 2023) | |
| 4 | CM/LB | Chlorogenic acid | C_16_H_18_O_9_ | 353.0878 | 353.0955 | [M-H]^-^ | 11.36 | 135.0479, 191.0489, 179.0403 | 135.0440, 179.0445, 191.0401 | (Ouyang, Fan, Wei, Chang & He, 2022) | |
| 5 | LB | DL-Arabinose | C_5_H_10_O_5_ | 149.0955 | 149.0998 | [M-H]^-^ | 14.30 | 58.8862, 130.4071, 78.6952 | 58.8000, 88.8000 | (Li et al., 2021) | |
| 6 | ZJ | Oleanolic acid | C_30_H_48_O_3_ | 455.2031 | 455.2015 | [M-H]^-^ | 15.64 | 150.9553, 429.0368, 59.0163 | - | (Guo et al., 2011) | |

Abbreviations: PL, *Puerariae* *lobata* (Willd.) Ohwi; LB, *Lycium barbarum* L.; HA, *Hovenia acerba* Lindl. seeds; CM, *Chrysanthemum morifolium* Ramat; CR, *Citri Reticulatae Pericarpium*; ZJ, *Zizyphus jujuba* Mill.

| **Table S2 Details about 81 serum components of GZS in ALI mouse** | | | | | | | | | | |
| --- | --- | --- | --- | --- | --- | --- | --- | --- | --- | --- |
| **NO.** | **Resourse** | **Name** | **Formula** | **Calculated MS**  **(m/z)** | **Measured MS**  **(m/z)** | **Ion Mode** | **Rt(min)** | **MS/MS Fragments** | **References**  **MS/MS** | **References** |
| 1 | PL | Puerarin | C_21_H_20_O_9_ | 417.1180 | 417.1173 | [M+H]^+^ | 7.58 | 297.0763, 267.0615, 321.0749 | 267.0653, 297.0758, 321.0758 | (Zhang et al., 2021) |
| 2 | PL | Vitexin 2"-O-β-D-glucopyranoside/isovitexin 2"-O-β-D-glucopyranoside | C_27_H_30_O_15_ | 593.1512 | 593.1584 | [M-H]^-^ | 51.10 | 473.0541, 209.0490, 165.0585 | 473.0505, 413.1399 | (Chen et al., 2022) |
| 3 | PL | 6-Hydroxygenistein-7-oglucoside | C_21_H_20_O_11_ | 447.0933 | 447.0985 | [M-H]^-^ | 10.37 | 285.0451, 284.9679 | 284.9692 | (Chen et al., 2022) |
| 4 | PL | Kakkatin | C_16_H_12_O_5_ | 283.0612 | 283.0682 | [M-H]^-^ | 9.55 | 268.0219, 252.5506 | 268.0292, 254.9701, 238.9880 | (Chen et al., 2022) |
| 5 | PL | Daidzein-4'-O-glucoside | C_21_H_20_O_9_ | 417.118 | 417.1173 | [M+H]^+^ | 8.90 | 256.0683, 255.0655, 316.0344 | 255.06492 | (Liu et al., 2018) |
| 6 | PL | Genistein-4'-O-glucoside | C_21_H_18_O_11_ | 447.0922 | 447.0972 | [M-H]^-^ | 14.65 | 271.0555, 242.0569 | 271.06097 | (Liu et al., 2018) |
| 7 | PL | Puerarin-6"-O-xyloside | C_26_H_28_O_13_ | 549.1603 | 549.1609 | [M+H]^+^ | 7.51 | 267.0628, 173.0889, 470.6787 | 295.0606, 267.0659 | (Zhang et al., 2021) |
| 8 | PL | 3'-Methoxypuerarin | C_22_H_22_O_10_ | 447.1286 | 447.1272 | [M+H]^+^ | 8.03 | 429.1123, 411.1013, 327.0846 | 429.11713, 411.10665, 297.07529 | (Liu et al., 2018) |
| 9 | PL | Mirificin | C_26_H_28_O_13_ | 549.1603 | 549.1547 | [M+H]^+^ | 7.71 | 417.1173, 400.1008, 382.0960 | 417.11721, 399.10673, 381.09615, 351.08566, 321.07521, 297.07532, 267.06486 | (Liu et al., 2018) |
| 10 | PL | Glycitin | C_22_H_22_O_10_ | 447.1286 | 447.1272 | [M+H]^+^ | 14.65 | 285.0736, 271.0555, 242.0569 | 285.0758, 270.0523 | (Zhang et al., 2021) |
| 11 | PL | Genistin | C_21_H_20_O_10_ | 433.1129 | 433.1148 | [M+H]^+^ | 11.54 | 271.0625, 272.0646, 71.0864 | 271.05989 | (Liu et al., 2018) |
| 12 | PL | Tectoridin | C_22_H_22_O_11_ | 463.1235 | 463.1219 | [M+H]^+^ | 12.04 | 298.9686, 301.0703 | 446.1283, 415.0985, 340.9573, 298.9627 | (Chen et al., 2022) |
| 13 | PL | Daidzein | C_15_H_10_O_4_ | 255.0652 | 255.0654 | [M+H]^+^ | 8.93 | 199.0993, 137.0243, 181.0644 | 137.5000, 199.1000 | (Shi, Li, Zhang, Fu & Zhang, 2015) |
| 14 | PL | Genistein | C_15_H_10_O_5_ | 269.0518 | 269.0513 | [M-H]^-^ | 12.47 | 270.0515, 225.0339, 201.0591 | 240.0180, 225.0333, 201.0156 | (Chen et al., 2022) |
| 15 | PL | Biochanin A | C_16_H_12_O_5_ | 285.0757 | 285.0710 | [M+H]^+^ | 14.71 | 270.0596, 215.4203, 290.1209 | 270.05181 | (Liu et al., 2018) |
| 16 | PL | 3'-Hydroxypuerarin | C_21_H_20_O_10_ | 433.1129 | 433.1148 | [M+H]^+^ | 6.51 | 283.0584, 397.2536, 313.0688 | 415.10166, 397.09101, 313.07003, 283.05955 | (Liu et al., 2018) |
| 17 | PL | Daidzin | C_21_H_20_O_9_ | 417.1180 | 417.1189 | [M+H]^+^ | 8.91 | 255.0663, 199.0751, 137.0208 | 255.06475 | (Liu et al., 2018) |
| 18 | PL/LB/HA | Kaempferol | C_15_H_10_O_6_ | 285.0405 | 285.0423 | [M-H]^-^ | 19.82 | 269.0002, 212.8675, 171.0595 | 269.0004, 257.0361, 240.9996 | (Chen et al., 2022) |
| 19 | PL | Ononin | C_22_H_22_O_9_ | 431.1337 | 431.1369 | [M+H]^+^ | 11.57 | 268.0824, 269.0480, 151.0030 | 269.08055 | (Liu et al., 2018) |
| 20 | PL | Glycitein | C_16_H_12_O_5_ | 285.0757 | 285.0778 | [M+H]^+^ | 0.57 | 213.0539, 137.0559 | 213.0546, 137.0234 | (Zhang et al., 2021) |
| 21 | PL | Tectorigenin | C_16_H_12_O_6_ | 301.0707 | 301.0755 | [M+H]^+^ | 27.46 | 255.0085, 283.9737, 241.1593 | 283.9724, 255.0026 | (Chen et al., 2022) |
| 22 | HA/CR | Dihydromyricetin | C_15_H_12_O_8_ | 319.0459 | 319.0521 | [M-H]^-^ | 8.72 | 193.0125, 125.0256, 111.0547 | 125.0246, 193.0148, 301.0359 | (Cheng, Sun, Hu, Deng, Zhang & Li, 2023) |
| 23 | HA | 3-Caffeoylquinic acid | C_16_H_18_O_9_ | 353.0378 | 353.0389 | [M-H]^-^ | 19.45 | 191.0554, 127.0778 | 191.0576 | (Peng, Deng, Chen, Sun, Zhang & Li, 2018) |
| 24 | HA | Apigenin | C_15_H_10_O_5_ | 269.0455 | 269.0413 | [M-H]^-^ | 12.48 | 239.0401, 180.0624 | 239.0335, 227.0335 | (Peng et al., 2018) |
| 25 | HA | Citric acid | C_6_H_8_O_7_ | 191.0197 | 191.0238 | [M-H]^-^ | 2.41 | 111.0082, 77.4889 | 57.0347, 111.0086 | (Cheng et al., 2023) |
| 26 | HA | Isoorientin-2"-O-rhamnoside | C_27_H_30_O_15_ | 593.1512 | 593.1520 | [M-H]^-^ | 51.45 | 431.0991, 277.2237 | 284.0331, 311.0567, 353.0676, 431.0982, 473.1099 | (Cheng et al., 2023) |
| 27 | HA | Myricetin | C_15_H_10_O_8_ | 317.0303 | 317.0313 | [M-H]^-^ | 27.45 | 317.0334, 277.0313 | 317.0335 | (Cheng et al., 2023) |
| 28 | HA | Dihydroquercetin | C_15_H_12_O_7_ | 303.0510 | 303.0589 | [M-H]^-^ | 36.38 | 285.0287, 163.0802, 114.4652 | 285.0290, 151.0020,  125.0232 | (Peng et al., 2018) |
| 29 | HA | Rutin | C_27_H_30_O_16_ | 609.1461 | 609.1446 | [M-H]^-^ | 11.49 | 301.0380, 286.0536, 271.1541 | 300.0264, 301.0337, 271.0243 | (Peng et al., 2018) |
| 30 | HA | Quercetin | C_15_H_10_O_7_ | 301.0367 | 301.0392 | [M-H]^-^ | 11.51 | 149.0295, 118.0255 | 105.0344, 149.0248 | (Cheng et al., 2023) |
| 31 | HA | Vitexin | C_21_H_20_O_10_ | 431.0984 | 431.1069 | [M-H]^-^ | 10.49 | 283.0686, 285.0474 | 117.0346, 283.0616, 311.0572, 344.0185 | (Cheng et al., 2023) |
| 32 | HA | Azelaic acid | C_9_H_16_O_4_ | 187.0976 | 187.1013 | [M-H]^-^ | 12.18 | 125.0904, 57.0367 | 57.0347, 125.0971 | (Cheng et al., 2023) |
| 33 | HA | Ursonic acid | C_29_H_42_O_4_ | 453.3010 | 453.2944 | [M-H]^-^ | 18.75 | 409.3128, 391.2738 | 409.3111, 391.2993, 363.2662 | (Peng et al., 2018) |
| 34 | HA | Vitexin-2"-O-glucoside | C_27_H_30_O_15_ | 593.1512 | 593.1416 | [M-H]^-^ | 23.13 | 383.0791, 322.9685 | 353.0676, 383.0790, 413.0882, 473.1099 | (Cheng et al., 2023) |
| 35 | HA | Hovenitins Ⅰ/Hovenitins Ⅱ | C_16_H_14_O_8_ | 333.0616 | 333.0670 | [M-H]^-^ | 5.86 | 289.0748, 272.7001 | 315.0516, 289.0718 | (Cheng et al., 2023) |
| 36 | CR | Neohesperidin | C_28_H_34_O_15_ | 611.1970 | 611.1961 | [M+H]^+^ | 11.54 | 303.0825, 112.0775 | 303.0859, 195.0291  177.0547, 153.0183 | (Sun et al., 2023) |
| 37 | CR | Cytosine | C_4_H_5_N_3_O | 112.0505 | 112.0504 | [M+H]^+^ | 1.77 | 95.0235, 71.0252, 55.9334 | 95.0240, 69.0447 | (Luo et al., 2023) |
| 38 | CR | Proline | C_5_H_9_NO_2_ | 116.0706 | 116.0705 | [M+H]^+^ | 1.49 | 70.0655, 68.0494, 60.9864 | 70.0651 | (Luo et al., 2023) |
| 39 | CR | Stachydrine | C_7_H_13_NO_2_ | 144.1019 | 144.1013 | [M+H]^+^ | 1.57 | 58.0650, 84.0808, 144.1010 | 58.0647 | (Luo et al., 2023) |
| 40 | CR | Pyroglutamic acid | C_5_H_7_NO_3_ | 130.0499 | 130.0456 | [M+H]^+^ | 1.21 | 102.0523, 84.0807, 70.0652 | 102.0549, 84.0444 | (Luo et al., 2023) |
| 41 | CR | Phenylalanine | C_9_H_11_NO_2_ | 166.0863 | 166.0857 | [M+H]^+^ | 3.81 | 120.0803, 107.0480, 103.0539 | 149.0597, 131.0491, 120.0808, 107.0491, 103.0542 | (Luo et al., 2023) |
| 42 | CR | trans-3-Indoleacrylic acid | C_11_H_9_NO_2_ | 188.0706 | 188.0699 | [M+H]^+^ | 5.93 | 170.0606, 146.0594, 118.0650 | 170.0600, 146.0600, 144.0808, 118.0651 | (Luo et al., 2023) |
| 43 | CR | Tryptophan | C_11_H_12_N_2_O_2_ | 205.0972 | 205.0972 | [M+H]^+^ | 6.04 | 188.0720, 159.0885, 118.0645 | 188.0706, 170.0600, 159.0917, 146.0600, 144.0808, 118.0651, 91.0543 | (Luo et al., 2023) |
| 44 | CR | Naringin | C_27_H_32_O_14_ | 579.1719 | 579.1742 | [M-H]^-^ | 10.85 | 273.0698, 272.0657, 151.0066 | 273.0759 | (Wang, Lin, Wang, Yang & Niu, 2024) |
| 45 | CR | Hesperidin | C_28_H_34_O_15_ | 611.1970 | 611.2034 | [M+H]^+^ | 11.49 | 431.1347, 414.1436, 358.6223 | 431.1379, 413.1202, 395.1131, 369.0974, 315.0864, 303.0857, 263.0544, 219.0281, 195.0285, | (Luo et al., 2023) |
| 46 | CR | Isosinensetin | C_20_H_20_O_7_ | 371.1136 | 371.1142 | [M-H]^-^ | 21.77 | 358.1039, 347.1892 | 358.1036, 343.0802, 329.0994 | (Luo et al., 2023) |
| 47 | CR | 4',5,6,7-Tetramethoxyflavanone | C_19_H_20_O_6_ | 343.1187 | 343.1149 | [M-H]^-^ | 29.09 | 330.1066, 303.1441, 161.1354 | 330.1085, 315.0856, 313.1049, 303.1221, 243.1351, 211.0597, 195.1012, 161.0595 | (Luo et al., 2023) |
| 48 | CR | Nobiletin | C_21_H_22_O_8_ | 403.1387 | 403.1381 | [M+H]^+^ | 21.38 | 388.1168, 373.0905, 341.0462 | 425.1211, 388.1153, 373.0923, 358.0673 | (Wang et al., 2024) |
| 49 | CR | 8-Hydroxy-3,5,6,7,3′,4′-hexamethoxyflavone | C_21_H_22_O_9_ | 417.1191 | 417.1266 | [M-H]^-^ | 11.69 | 404.1174, 162.8409, 121.0299 | 404.1103, 389.0869, 386.1012, 374.0627,  371.0759, | (Wang et al., 2024) |
| 50 | ZJ | Catechin | C_15_H_14_O_6_ | 289.0718 | 289.0715 | [M-H]^-^ | 26.27 | 125.0276, 109.9714, 97.5134 | 109.0282, 123.0439, 97.0281, 125.0230 | (Qin et al., 2022) |
| 51 | ZJ | (15Z)-9,12,13-trihydroxy-15-octadecenoic acid | C_18_H_34_O_5_ | 329.2334 | 329.2329 | [M-H]^-^ | 37.18 | 265.1537, 201.1107, 27.5006 | 171.1018, 201.1126, 127.1117 | (Qin et al., 2022) |
| 52 | ZJ | Coumaroylhexose | C_15_H_18_O_8_ | 325.0929 | 325.0925 | [M-H]^-^ | 27.49 | 183.0612, 119.0432, 79.9601 | 119.0488, 163.0388 | (Qin et al., 2022) |
| 53 | ZJ | Ferulic acids-O-glucoside | C_16_H_20_O_9_ | 355.1035 | 355.1032 | [M-H]^-^ | 38.73 | 205.1204, 193.0448, 125.5498 | 134.0360, 193.0496, 149.0595, 178.0261 | (Qin et al., 2022) |
| 54 | ZJ | Ferulic acid-O-derivative | C_23_H_32_O_16_ | 563.1618 | 563.1613 | [M-H]^-^ | 9.28 | 193.0448, 109.5185, 55.8047 | 193.0496, 175.0390, 355.1025, 134.0361 | (Qin et al., 2022) |
| 55 | ZJ | Isoliquiritin isomer | C_21_H_22_O_9_ | 417.1191 | 417.1187 | [M-H]^-^ | 11.86 | 135.0049, 121.0299 | 255.0661, 119.0490, 135.0076, 153.0183 | (Qin et al., 2022) |
| 56 | ZJ | Methylation of magnoflorine | C_21_H_27_NO_4_ | 358.2013 | 358.2023 | [M+H]^+^ | 35.09 | 253.0854, 106.0854, 58.0728 | 58.0660, 253.0862, 313.1073 | (Qin et al., 2022) |
| 57 | ZJ | Naringenin-C-glucoside | C_21_H_22_O_10_ | 433.1140 | 433.1135 | [M-H]^-^ | 13.39 | 313.0705, 119.0518, 177.0736 | 313.0716, 271.0611,  343.0819 | (Qin et al., 2022) |
| 58 | ZJ | Catechin-O-glucoside | C_21_H_24_O_11_ | 451.1246 | 451.1243 | [M-H]^-^ | 8.99 | 245.0806, 125.0218, 79.5639 | 245.0815, 289.0716,  109.0282, 125.0231 | (Qin et al., 2022) |
| 59 | ZJ | Hydroxylation of coclaurine | C_17_H_19_NO_4_ | 302.1387 | 302.1396 | [M+H]^+^ | 21.85 | 123.0466, 107.0845, 99.0488 | 123.0446, 143.0496, 175.0759, 285.1130 | (Qin et al., 2022) |
| 60 | ZJ | Hydroxypalmitic acid | C_16_H_32_O_3_ | 271.2273 | 271.2277 | [M-H]^-^ | 34.35 | 253.2129, 225.2271, 79.4821 | 225.2217, 253.2169 | (Qin et al., 2022) |
| 61 | ZJ | Hyperoside | C_21_H_20_O_12_ | 463.0882 | 463.0880 | [M-H]^-^ | 32.23 | 271.0248, 255.0224, 78.9617 | 300.0273, 301.0352,  271.0246, 255.0296 | (Qin et al., 2022) |
| 62 | CM/LB | Chlorogenic acid | C_16_H_18_O_9_ | 353.0878 | 353.0951 | [M-H]^-^ | 6.92 | 191.0590, 179.0420, 126.9082 | 135.0440, 179.0445, 191.0401 | (Ouyang, Fan, Wei, Chang & He, 2022) |
| 63 | CM | Cynaroside | C_21_H_20_O_11_ | 449.1078 | 449.1082 | [M+H]^+^ | 10.38 | 285.0459, 151.0164, 114.0904 | 285.04050, 151.0037, 133.0295 | (Ouyang et al., 2022) |
| 64 | CM | Luteolin | C_15_H_10_O_6_ | 287.0550 | 287.0525 | [M+H]^+^ | 19.75 | 213.0537, 199.0748 | 269.0440, 241.0493, 213.0548, 153.0183 | (Li et al., 2023) |
| 65 | CM | Acacetin | C_16_H_12_O_5_ | 283.0612 | 283.0666 | [M-H]^-^ | 14.69 | 268.0433, 240.0473, 195.0450 | 268.0376, 240.0426, 212.0476, 195.0450 | (Li et al., 2023) |
| 66 | CM | Isochlorogenic acid A | C_25_H_24_O_12_ | 515.1194 | 515.1193 | [M-H]^-^ | 11.38 | 353.0852, 191.0530, 390.9024 | 353.0852, 191.0528 | (Ouyang et al., 2022) |
| 67 | CM | Isochlorogenic acid B | C_25_H_24_O_12_ | 515.1195 | 515.1195 | [M-H]^-^ | 11.41 | 353.0857, 192.0642, 191.0475 | 353.0856, 191.0496 | (Ouyang et al., 2022) |
| 67 | CM | Isochlorogenic acid C | C_25_H_24_O_12_ | 515.1195 | 515.1196 | [M-H]^-^ | 11.41 | 353.0854, 192.0642, 191.0515 | 353.0855, 191.0512 | (Ouyang et al., 2022) |
| 68 | CM | Apigenin-7-O-glucuronic acid | C_21_H_18_O_11_ | 445.0776 | 445.0777 | [M-H]^-^ | 10.82 | 271.0527, 221.1005, 203.0182 | 271.0584, 269.0438 | (Ouyang et al., 2022) |
| 69 | CM | Luteolin-7-O-rutinoside | C_27_H_30_O_15_ | 593.1512 | 593.1520 | [M-H]^-^ | 22.61 | 285.1562, 257.0206 | 285.1541, 257.0201, 152.0378 | (Dai & Sun, 2022) |
| 70 | CM | Isorhamnetin | C_16_H_12_O_7_ | 317.0656 | 317.0667 | [M+H]^+^ | 26.24 | 300.0190, 109.0642, 182.8915 | 300.0112, 287.0037,  272.0724, 151.0633 | (Dai et al., 2022) |
| 71 | CM | Baicalin | C_21_H_18_O_11_ | 445.0776 | 445.0713 | [M-H]^-^ | 17.97 | 269.0432, 192.1304, 167.0307 | 269.0432, 192.1304, 167.0307 | (Ouyang et al., 2022) |
| 72 | CM | Diosmetin | C_16_H_12_O_6_ | 301.0707 | 301.0756 | [M+H]^+^ | 27.47 | 229.0482, 172.1192 | 286.0470, 258.0521,229.0494, 153.0183 | (Li et al., 2023) |
| 73 | CM | Apigetrin | C_21_H_20_O_10_ | 431.0984 | 431.1069 | [M-H]^-^ | 10.40 | 284.8758, 175.1327, 151.0204 | 269.0201, 175.1327,  151.0204 | (Dai et al., 2022) |
| 74 | CM | Bruceantin | C_28_H_36_O_11_ | 549.2330 | 549.2309 | [M+H]^+^ | 7.97 | 347.1863, 352.0900 | 347.1867, 329.1759 | (Ouyang et al., 2022) |
| 75 | CM | Chrysin | C_15_H_10_O_4_ | 253.0506 | 253.0555 | [M-H]^-^ | 13.82 | 176.0494, 126.9622, 77.0224 | 151.0199, 101.0028,  176.0433, 77.0285 | (Dai et al., 2022) |
| 76 | CM | Cryptochlorogenic acid | C_16_H_18_O_9_ | 353.0878 | 353.0938 | [M-H]^-^ | 6.86 | 191.0598, 133.3921 | 173.0435, 179.0371, 191.0508 | (Ouyang et al., 2022) |
| 77 | LB | Ferulic acid | C_10_H_10_O_4_ | 193.0506 | 193.0538 | [M-H]^-^ | 7.41 | 134.0348, 108.3439, 60.976 | 134.0379 | (Zhang et al., 2018) |
| 78 | LB | Dihydrocaffeoyl spermidine derivative | C_41_H_57_O_20_N_3_ | 912.3608 | 912.3593 | [M+H]^+^ | 38.32 | 750.3065, 588.2751, 310.2121 | 750.3066, 634.2957, 472.2431, 310.2121, 220.0965, 163.0398 | (Xiao et al., 2019) |
| 79 | LB | Ellagic acid | C_14_H_6_O_8_ | 300.9990 | 300.0031 | [M-H]^-^ | 29.46 | 217.0015, 122.3052 | 217.0034, | (Zhang et al., 2018) |
| 80 | LB | veratronic acid | C_9_H_10_O_4_ | 181.0506 | 181.0506 | [M-H]^-^ | 6.13 | 176.1570, 137.0664 | 137.0605 | (Zhang et al., 2018) |
| 81 | LB | Lycibarbarphenylpropanoid F isomer | C_23_H_32_O_13_ | 515.1770 | 515.1790 | [M-H]^-^ | 11.89 | 191.0565, 353.0830 | 395.0988, 353.0877, 274.9858 191.0557, 161.0242 | (Xiao et al., 2019) |

Abbreviations: PL, *Puerariae* *lobata* (Willd.) Ohwi; LB, *Lycium barbarum* L.; HA, *Hovenia acerba* Lindl. seeds; CM, *Chrysanthemum morifolium* Ramat; CR, *Citri Reticulatae Pericarpium*; ZJ, *Zizyphus jujuba* Mill.

| **Tab. S3 Differential metabolites** | | | | | | | |
| --- | --- | --- | --- | --- | --- | --- | --- |
| **Metabolite** | **RT/min** | **m/z** | **Formula** | **VIP** | **Model *vs* Control** | **Treatment *vs* Model** | **Metabolic pathway** |
| Acetylcysteine | 1.585 | 164.0378 | C_5_H_9_NO_3_S | 1.06 | ↓* | ↑** | - |
| Hippuric acid | 5.501 | 180.0663 | C_9_H_9_NO_3_ | 0.87 | ↓ | ↑** | Phenylalanine metabolism |
| Estrone | 7.098 | 315.1571 | C_18_H_22_O_2_ | 0.17 | ↓ | ↑* | Steroid hormone biosynthesis; Ovarian steroidogenesis; Prolactin signaling pathway |
| 2,5-Furandicarboxylic acid | 2.121 | 139.0028 | C_6_H_4_O_5_ | 1.95 | ↑ | ↑ | - |
| LPA 18:0 | 10.320 | 437.2665 | C_21_H_43_O_7_P | 0.32 | ↑** | ↓** | Glycerolipid metabolism; Glycerophospholipid metabolism; Biosynthesis of secondary metabolites; Phospholipase D signaling pathway; Gap junction; Fat digestion and absorption; Vitamin digestion and absorption; Pathways in cancer |
| PC 38:6 | 11.177 | 828.5520 | C_46_H_80_NO_8_P | 0.84 | ↑ | ↓ | - |
| LPI 20:3 | 9.592 | 621.3052 | C_29_H_51_O_12_P | 0.88 | ↑ | ↓ | - |
| LPE 17:1 | 9.103 | 464.2774 | C_22_H_44_NO_7_P | 0.60 | ↑ | ↓ | - |
| LPE 19:1 | 10.002 | 492.3093 | C_24_H_48_NO_7_P | 0.75 | ↑ | ↓ | - |
| PE 18:2_18:2 | 11.247 | 740.5230 | C_41_H_74_NO_8_P | 0.88 | ↑ | ↓ | - |
| CAR 18:2 | 7.899 | 424.3414 | C_25_H_46_NO_4_ | 1.27 | ↑ | ↑ | - |
| CAR 20:3 | 8.177 | 450.3569 | C_27_H_48_NO_4_ | 1.10 | ↓ | ↑** | - |
| CAR 22:6 | 7.808 | 472.3415 | C_29_H_46_NO_4_ | 0.62 | ↓ | ↑ | - |
| CAR 20:4 | 7.854 | 448.3415 | C_27_H_46_NO_4_ | 1.28 | ↓ | ↑** | - |
| Corey Lactone Diol | 1.474 | 173.0809 | C_8_H_12_O_4_ | 0.84 | ↓ | ↑* | - |
| 23-Nordeoxycholic acid | 7.506 | 377.2699 | C_23_H_38_O_4_ | 0.65 | ↓ | ↑ | - |
| Monobenzyl phthalate | 5.641 | 255.0663 | C_15_H_12_O_4_ | 0.45 | ↓* | ↑** | - |
| LPC 19:1-SN1 | 10.079 | 536.3709 | C_27_H_54_NO_7_P | 1.08 | ↑ | ↓ | - |

| **Tab. S4 Metabolomics combined with network pharmacology ranking** | | | | | | |
| --- | --- | --- | --- | --- | --- | --- |
| **Ranking** | **Ingredients** | **Natural food** | **Number of targets** | **Joint analysis of targets** | **Network pharmacology targets** | **Network Pharmacology Ranking** |
| 1 | luteolin | CM | 8 | IL6、SRC、CASP3、BCL2、PPARG、TNF、TP53、MMP9 | ABCB1、ACHE、ADORA1、ADORA2A、AHR、AKR1A1、ALOX5、APP、ARG1、BIRC5、CA4、CA6、CASP3、CASP7、CASP9、CCND1、CD40LG、CDK1、CDK2、CDK4、CDK6、CFTR、CXCR1、DPP4、EGF、EGFR、ERBB2、ESR1、ESR2、F2、GSK3B、HGF、HMOX1、ICAM1、IFNG、IGF1R、IL10、IL2、IL4、IL6、INSR、KDR、MAOA、MAPK1、MDM2、MMP1、MMP13、MMP2、MMP3、MMP9、MPO、PARP1、PCNA、PIK3CG、PLA2G1B、PLG、PLK1、PPARG、PTGS1、PTGS2、PTK2、RB1、SRC、TNF、TOP2A、TP53、TTR、VEGFA、XDH | 4 |
| 2 | Quercetin | HA/ZJ | 7 | IL6、CASP3、BCL2、PPARG、TNF、TP53、MMP9 | ACHE、ADRB、AHR、ALOX5、BAX、BCL2、BIRC5、CASP3、CASP8、CASP9、CAV1、CCL2、CCND1、CD40LG、CDKN2A、CHEK2、COL1A1、CRP、CXCL10、CXCL2、CYP1A1、CYP1A2、CYP3A4、DIO1、DPP4、EGF、ERBB2、F10、F2、F3、F7、FOS、GJA1、GSTM1、HIF1A、HMOX1、HSPB1、ICAM1、IFNG、IGF2、IL10、IL1A、IL2、IL6、IL8、INSR、IRF1、MAOB、MAPK1、MMP1、MMP2、MMP3、MMP9、MPO、MYC、NFE2L2、NOS2、NOS3、NQO1、NR1I2、ODC1、PARP1、PIK3CG、PLAT、PLAU、PON1、POR、PPARA、PPARG、PRKCA、PTGS1、PTGS2、RAF1、RASSF1、RB1、SOD1、STAT1、TGFB1、TNF、TOP2A、TP53、VEGFA、XDH | 2 |
| 3 | Kaempferol | LB/HA/PL | 6 | CASP3、BCL2、PPARG、TNF、SRC、MMP9、 | ESR1、ESR2、PTK2、GSK3、IGF1R、CXCR1、MMP13、MMP2、MMP3、MMP9、MAPT、MAOA、MPO、ABCB1、PIK3CG、PLA2G1B、PARP1、PTGS1、PLK1、F2、TTR、SRC、KDR、HSP90AA1 | 1 |
| 4 | Genistein | PL | 6 | CASP3、STAT3、BCL2、PPARG、TP53、MMP9 | ABCB1、ACHE、ADORA1、ADORA2A、ALDH2、ALOX5、APOA1、APP、ARG1、ATM、BAX、BCL2、BIRC5、CA4、CA6、CASP3、CASP9、CCL2、CDC20、CDK2、CDK6、CDKN2A、CFTR、CHEK1、CHEK2、CRHR1、CRYAB、EGF、EGFR、ERBB2、ESR1、ESR2、ESRRB、F1、FN1、FOS、GFAP、GSK3B、HIF1A、HMGCR、ICAM1、IGF1R、IL2、IL8、INS、KLK3MAOA、MAOBMAPK1、MAPK12、MAPK14、MDM2、MIF、MMP2、MMP9、MMP9、NOS2、NOS3、PARP1、PIK3CG、PLAT、PLAU、PLK1、PON1、PPARA、PPARG、PTGS1、PTGS2、RAF1、SOD2、STAT1、STAT3、TGFB1、TIMP、TNF、TP53、TTR、VEGFA、XDH | 3 |
| 5 | Apigenin | HA | 6 | SRC、CASP3、BCL2、TNF、TP53、MMP9 | ABCB1、ACHE、ADORA1、ADORA2A、AHR、ALOX5、APC、APP、ARG1、BAX、BCL2、CA4CA6、CASP3、CASP9、CCND1、CD40LG、CDK1、CDK2、CDK4、CDK6、CDKN2A、CFTR、CXCR1、DPP4、EEF1E1、EGFR、ESR1、ESR2、F10、F2、F7、FOS、GSK3B、HIF1A、HMOX1、ICAM1、IFNG、IGF1R、IKBKG、IL2、IL4、INS、INSR、KDR、MAOA、MDM2、MMP1、MMP13、MMP2、MMP3、MMP9、MPG、MPO、ODC1、PARP1、PIK3CG、PLAU、PLG、PLK1、PTGS1、PTGS2、PTK2、RB1、SRC、TNF、TP53、TTR、VEGFA、XDH | 5 |
| 6 | Myricetin | HA | 6 | IL6、SRC、STAT3、PPARG、TNF、MMP9 | ABCB1、ACHE、ADORA1、ADORA2A、AHR、AKR1A1、ALOX5、APEX1、APP、ARG1、BAX、CA4、CA6、CASP3、CASP8、CASP9、CD36、CDK1、CDK2、CDK6、CXCR1、DPP4、EGFR、ESR2、F2、FYN、GSK3B、IGF1R、IL6、INSR、JAK1、KDR、MAOA、MAPT、MMP13、MMP2、MMP3、MMP9、MPG、MPO、MYLK、PARP1、PIK3CG、PLA2G1B、PLK1、PPARG、PSMD4、PTGS1、PTGS2、PTK2、SRC、STAT3、TH、TNF、TOP2A、TTR、XDH | 6 |
| 7 | Puerarin | PL | 6 | CASP3、STAT3、BCL2、PPARG、TNF、MMP9 | BAX、BCL2、BIRC5、CASP3、CASP8、CASP9、CDKN1B、CHEK2、ESR1、FOS、GSK3B、HIF1A、LEPR、MMP2、MMP9、NOS2、NOS3、PLAT、PPARG、PRKCA、PTGS2、SELP、SOD1、STAT3、JUN、TNF、VEGFA | 22 |
| 8 | Daidzein | PL | 5 | IL6、CASP3、PPARG、TNF、TP53 | ABCB1、ACHE、ADORA1、ADORA2A、ADRB2、、HR、ALDH2、ALOX5、APOB、BARD1、BAX、CA4、CASP3、CAT、CAV1、CDK2、CDKN1A、CHEK1、CYP3A4、ECE1、EGFR、ESR1、ESR2、ESRRB、F10、FOS、GADD45A、GH1、HSP90AA1、ICAM1、IGF1、IGF1R、IL2、IL4、IL6、MAOA、MAOB、MAPK14、MIF、MKI67、MT2A、NOS2、NOS3、PLAT、PLAU、PON1、PPARA、PPARG、PTGS1、PTGS2、RAD51、RHOA、SOD2、STAT1、JUN、TNF、TP53、VEGFA、XDH | 7 |
| 9 | Acacetin | CM | 5 | SRC、CASP3、BCL2、TP53、MMP9 | ABCB1、ACHE、ADORA1、ADORA2A、ADRB2、AHR、ALOX5、APP、ARG1、BAX、BCL2、CA4、CA6、CASP3、CASP8、CDK1、CDK2、CDK6、CFT、CHEK1、CDKN3、CYP1A1、CYP1A2、DPP4、EGFR、ESR1、ESR2、F2、FASN、GSK3B、IGF1R、KDR、MAOA、MAOB、MMP13、MMP2、MMP3、MMP9、MPG、MPO、NOS2、PARP1、PIK3CG、PLA2G2A、PLG、PLK1、PTGS1、PTGS2、PK2、SRC、TP53、TTR、XDH | 9 |
| 10 | Dihydromyricetin | HA/CR | 4 | SRC、BCL2、PPARG、MMP9 | ABCB1、ACHE、ADORA1、APP、BCL2、CA4、CA6、CASP3、ESR1、ESR2、FGFR1、HIF1A、KDR、MAOB、MAPK14、MAPT、MMP13、MMP14、MMP2、MMP9、PGD、PIK3CG、PLA2G1B、PLA2G2A、POLB、PPARA、PPARG、PTGS1、PTGS2、SRC、STAT1、TOP2A、VEGFA | 17 |
| 11 | Nobiletin | CR | 4 | BCL2、PPARG、TP53、MMP9 | ADORA1、ADORA2A、ALOX5、BAX、BCL2、CASP9、CD163、CHEK1、CREB1、DPP4、EPHB2、ESR2、F10、F2、F7、GSK3B、MMP9、NOS2、PPARG、PTGS1、PTGS2、TIMP1、TP53 | 23 |
| 12 | Isorhamnetin | CM | 3 | SRC、PPARG、MMP9 | ABCB1、ACHE、ADORA1、ADORA2A、AHR、AKR1A1、ALOX5、APEX1、APP、ARG1、CA4、CA6、CCNA2、CDK1、CDK2、CDK6、CHEK1、CXCR1、DPP4、EGFR、ESR2、F2、GSK3B、IGF1R、INSR、KDR、MAOA、MAPK14、MAPT、MMP13、MMP2、MMP3、MMP9、MPG、MPO、MYLK、NOS2、OLR1、PARP1、PIK3CG、PLA2G1B、PLG、PLK1、PPARG、PTGS1、PTGS、PTK2、SRC、TOP2A、TTR、XDH | 8 |
| 13 | Isosinensetin | CR | 3 | SRC、PPARG、MMP9 | PTGS2、ABCB1、ACHE、ADORA1、ADORA2A、ADRB2、ALOX5、APEX1、APP、ARG1、CA4、CA6、CDK1、CDK2、CHEK1、CYP1A1、CYP1A2、DPP4、EGFR、ESR2、F10、F2、F7、GSK3B、IGF1R、INSR、KDR、MAOA、MMP13、MMP2、MMP3、MMP9、NOS2、ODC1、PARP1、PDPK1、PIK3CA、PIK3CG、PLA2G1B、PLA2G2A、PLG、PLK1、PPARG、PTGS1、PTK2、SRC、XDH | 11 |
| 14 | Rutin | LB | 3 | IL6、CASP3、TNF | ACHE、ADORA1、ALOX5、C5AR1、CA4、CASP3、CAT、DIO1、HMGCR、IL2、IL6、IL8、INS、POR、PRKCA、PTGS2、RPS6K、A3、SOD1、TNF、XDH | 24 |
| 15 | Ononin | PL | 3 | SRC、PPARG、TNF | ABL1、ADORA1、ALDH2、DPP4、ESR1、F2、F7、IL2、KDR、MAPK14、MGMT、NOS2、PPARG、PTGS2、SRC、TNF、XDH | 30 |
| 16 | Chrysin | CM | 2 | SRC、MMP9 | ABCB1、ACHE、ADORA1、ADORA2A、AHR、ALOX5、APEX1、APP、ARG1、CA4、CDK1、CDK6、CFTR、CYP1A1、CYP1A2、DPP4、EGFR、ESR1、ESR2、F2、GSK3B、IGF1R、IL4、INSR、KDR、MAOA、MAOB、MAPT、MMP2、MMP9、MYLK、NOS2、NTRK2、PARP1、PIK3CG、PLA2G2A、PLG、PLK1、PRKDC、PTGS1、PTGS2、SLC6A4、SRC、TGFB1、TOP2A、TTR、XDH | 10 |
| 17 | Diosmetin | CM | 2 | SRC、MMP9 | ABCB1、ACHE、ADORA1、ADORA2A、AKR1A1、ALOX5、APP、ARG1、CA4、CA6、CDK1、CDK2、CDK6、CFTR、CXCR1、DPP4、EGFR、ESR1、ESR2、F2、GSK3B、IGF1R、KDR、MAOA、MMP13、MMP2、MMP3、MMP9、MPO、NOS2、PARP1、PLA2G1B、PLA2G2A、PLG、PLK1、PTGS1、PTGS2、PTK2、SRC、TTR、XDH | 12 |
| 18 | Ferulic acid | LB | 2 | STAT3、MMP9 | ABCB1、ACE、ADRB2、AHR、ALOX5、APP、CA4、CA6、CTNNB1、CYP1A1、CYP1A2、EGFR、ESR2、F2、F3、FYN、HSD11B1、MAOA、MAOB、MMP1、MMP2、MMP9、NFE2L2、NOS2、PARP1、PLAU、PRKCE、PTGS1、PTGS2、REN、SLC6A3、STAT3、TLR4、TOP2A、TTR | 13 |
| 19 | (15Z)-9,12,13-trihydroxy-15-octadecenoic acid | ZJ | 2 | CASP3、MAPK3 | ACP1、ADRB2、CASP3、CDK1、CDK2、ESR2、FAAH、FABP3、G6PD、HMGCR、HRH1、HSD11B1、INSR、LTB4R、MAPK10、MAPK14、MAPK3、MMP1、MMP13、MMP3、MMP8、NR1H3、NR3C1、NTRK2、PLA2G1B、POLB、PPARA、PPARG、PRKCA、PTGS1、PTGS2、PTPRF、S1PR1、SERPINA6、SLC22A6、SLC6A4、SOAT1 | 14 |
| 20 | Ursonic acid | HA | 2 | PPARG、MAPK3 | ACP1、ALOX5、CNR1、CYP2C19、ESR1、ESR2、FAAH、FABP3、G6PD、GRIK2、HMGCR、HSD11B1、LTB4R、MAPK3、MDM2、MME、MMP2、MMP3、NOS2、NR1H3、NR1H4、NR3C1、PLA2G1B、POLB、PPARA、PPARG、PSEN1、PTGS1、PTGS2、PTPN11、PTPRF、SERPINA6、SLC6A3、TOP2A | 16 |
| 21 | Tectorigenin | PL | 2 | BCL2、PPARG | ABCB1、ACHE、ADORA1、ADORA2A、ALDH2、CA4、CCNA2、CDK2、CHEK1、EGFR、ESR1、ESR2、ESRRB、F10、GSK3B、IGF1、IL2、MAOA、MAOB、MAPK14、MIF、NOS2、PIK3CG、PLAU、PON1、PPARA、PPARG、PTGS1、PTGS2、XDH | 19 |
| 22 | Ellagic acid | LB | 2 | SRC、MMP9 | ALOX5、CA4、CA6、CDK2、EGFR、ERBB2、ESR1、ESR2、GSK3B、GSR、GSTA1、GSTA2、GSTM1、HSPA1A、IGF1R、IGF2、IL8、INSR、KDR、LYN、MAOA、MMP2、MMP9、PLK1、PRKCA、PTGS2、PTK2、SRC、VEGFA、XDH | 20 |
| 23 | Daidzin | PL | 2 | PPARG、TNF | ACHE、ADORA1、ALDH2、DPP4、F10、F2、F7、GSK3B、HSP90AA1、IL2、KDR、MAOA、PPARG、PTGS2、TNF、XDH | 29 |
| 24 | Luteolin-7-O-rutinoside | CM | 2 | TNF、TP53 | ACHE、ADORA1、ALDH2、ALOX5、CA4、EGFR、IL2、PTGS2、RPS6KA3、TNF、TP53、XDH | 36 |
| 25 | Kakkatin | PL | 1 | PPARG | ABCB1、ACHE、ADORA1、ADORA2A、ADRB2、ALDH2、ALOX5、CA4、CCNA2、CDK2、CHEK1、CHRNA7、CTSL、EGFR、ESR1、ESR2、ESRRB、GSK3B、HDAC1、IL2、MAOA、MAOB、MAPK14、MIF、MMP1、NOS2、PON1、PPARA、PPARG、PTGS1、PTGS2、RAF1、SLC9A1、XDH | 15 |
| 26 | Glycitein | PL | 1 | PPARG | ABCB1、ACHE、ADORA1、ADORA2A、ALDH2、APP、CA4、CCNA2、CDK2、CHEK1、EGFR、ESR1、ESR2、ESRRB、GSK3B、HSP90AA1、IL2、MAOA、MAOB、MAPK14、MIF、MMP13、MMP8、NOS2、PON1、PPARA、PPARG、PTGS1、PTGS2、XDH | 18 |
| 27 | Biochanin A | PL | 1 | PPARG | ABCB1、ACHE、ADORA1、ADORA2A、ADRB2、ALDH2、CA4、CCNA2、CDK2、CHEK1、DPP4、EGFR、ESR1、ESR2、ESRRB、G、K3B、IL2、MAOA、MAOB、MAPK14、MIF、NOS2、PON1、PPARA、PPARG、PTGS1、PTGS2、XDH | 21 |
| 28 | Bruceantin | CM | 1 | JUN | ADORA2A、CCR1、CDK1、CDK2、FLT1、GSTM1、JUN、MMP1、MMP14、MMP3、MMP8、PAK1、PARP1、PRKCA、PRKCD、PRKCE、PRKCG、PTGS2、SLC2A1 | 26 |
| 29 | Chlorogenic acid | CM | 1 | CASP3 | ABCB1、APP、CASP3、CASP7、CASP8、ECE1、ELANE、KDR、MMP13、MMP2、PRKCA、PRKCD | 27 |
| 30 | Isochlorogenic acid B | CM | 1 | CASP3 | APP、CA4、CASP3、CASP7、CASP8、ELANE、FYN、HK1、MMP1、MMP13、MMP2、MMP8、PRKCA、PRKCD、SELL、SELP、TTR | 28 |
| 31 | Cynaroside | CM | 1 | TNF | ACHE、ADORA1、ALDH2、ALOX5、CA4、EGFR、IL2、NOS2、PLG、PTGS2、RPS6KA3、TNF、XDH | 33 |
| 32 | Tryptophan | CR | 1 | MMP9 | CTSL、EPHA2、EPHB2、MAOB、MIF、MME、MMP3、MMP9、MPO、PTGS1、PTGS2、SLC15A1、SLC6A3、SLC6A4 | 34 |
| 33 | Hyperoside | ZJ | 1 | TNF | ACHE、ADORA1、ALOX5、CA4、IL2、NOS2、PIK3CG、PTGS1、PTGS2、RPS6KA3、TNF、XDH | 35 |
| 34 | Hesperidin | CR | 1 | CASP3 | ABCB1、ADORA1、BAX、CA4、CA6、CASP3、EPHX2、ICAM1、MMP13、MMP8、PTGS1、PTGS2 | 39 |
| 35 | 3-Caffeoylquinic acid | HA | 1 | CASP3 | ABCB1、APP、CASP3、CASP7、CASP8、ECE1、ELANE、KDR、MMP13、MMP2、PRKCA、PRKCD | 40 |
| 36 | Naringin | CR | 1 | TNF | ADORA1、ADORA2A、EPHX2、MAOB、MMP1、MMP13、MMP8、PARP1、PTGS1、RAF1、TNF | 41 |
| 37 | Baicalin | CM | 1 | TNF | ACHE、ADORA1、ALDH2、EGFR、F10、IL2、PTGS2、RPS6KA3、TNF、XDH | 43 |
| 38 | Citric acid | HA | 1 | SRC | CHRNA7、F10、GRIA2、GRIK2、HDC、HMGCR、NOS2、PTGS2、SLC22A6、SRC | 45 |
| 39 | Glycitin | PL | 1 | TNF | ADORA1、ALDH2、F2、F7、HSP90AA1、IL2、PTGS2、TNF、XDH | 46 |
| 40 | Tectoridin | PL | 1 | TNF | ADORA1、ALDH2、HSP90AA1、IL2、MMP2、TNF、XDH | 49 |

Abbreviations: PL, *Puerariae* *lobata* (Willd.) Ohwi; LB, *Lycium barbarum* L.; HA, *Hovenia acerba* Lindl. seeds; CM, *Chrysanthemum morifolium* Ramat; CR, *Citri Reticulatae Pericarpium*; ZJ, *Zizyphus jujuba* Mill.


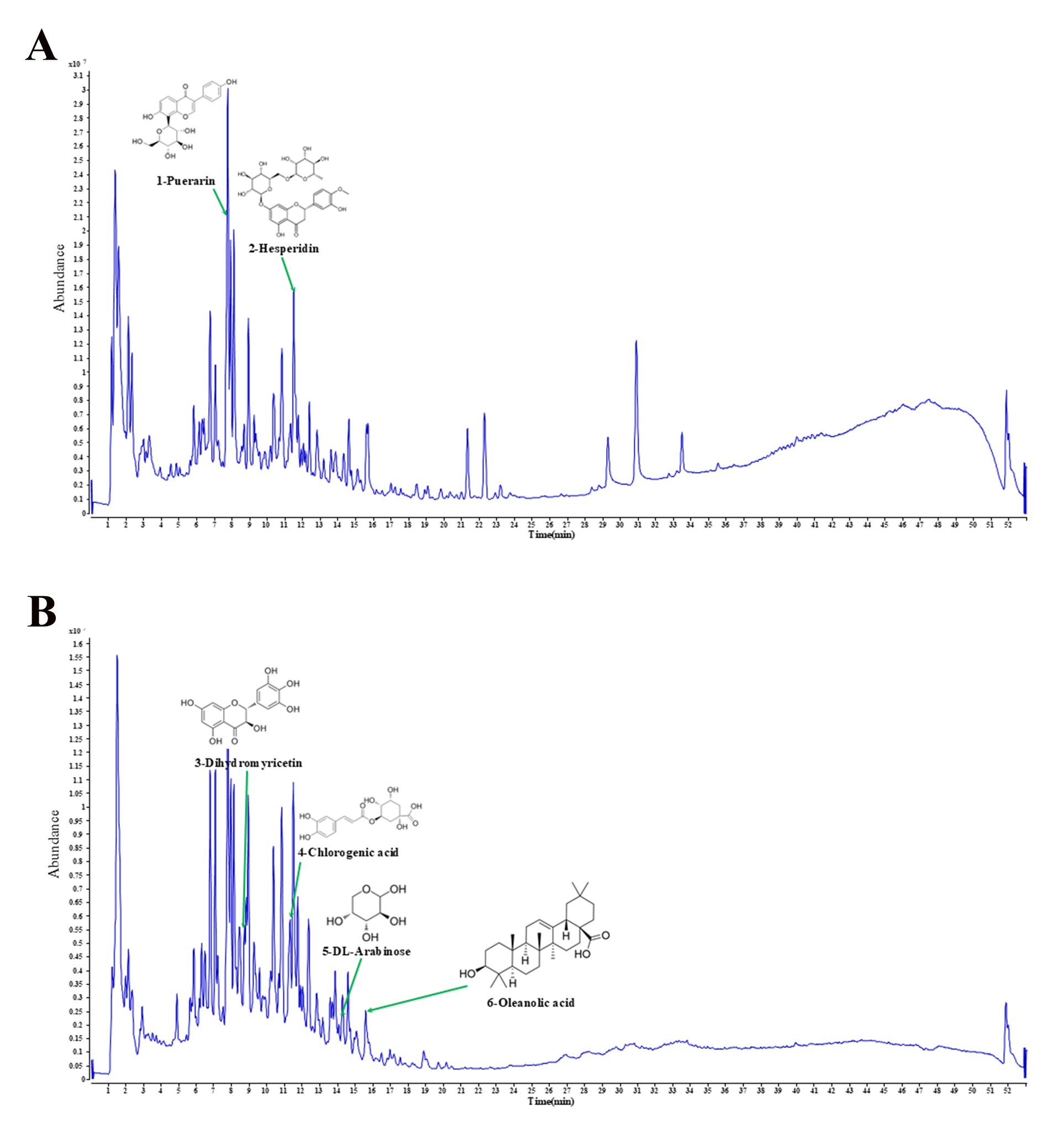


**Fig. S1**: Identification of natural food key components. **A**-positive mode, **B**-negative mode.


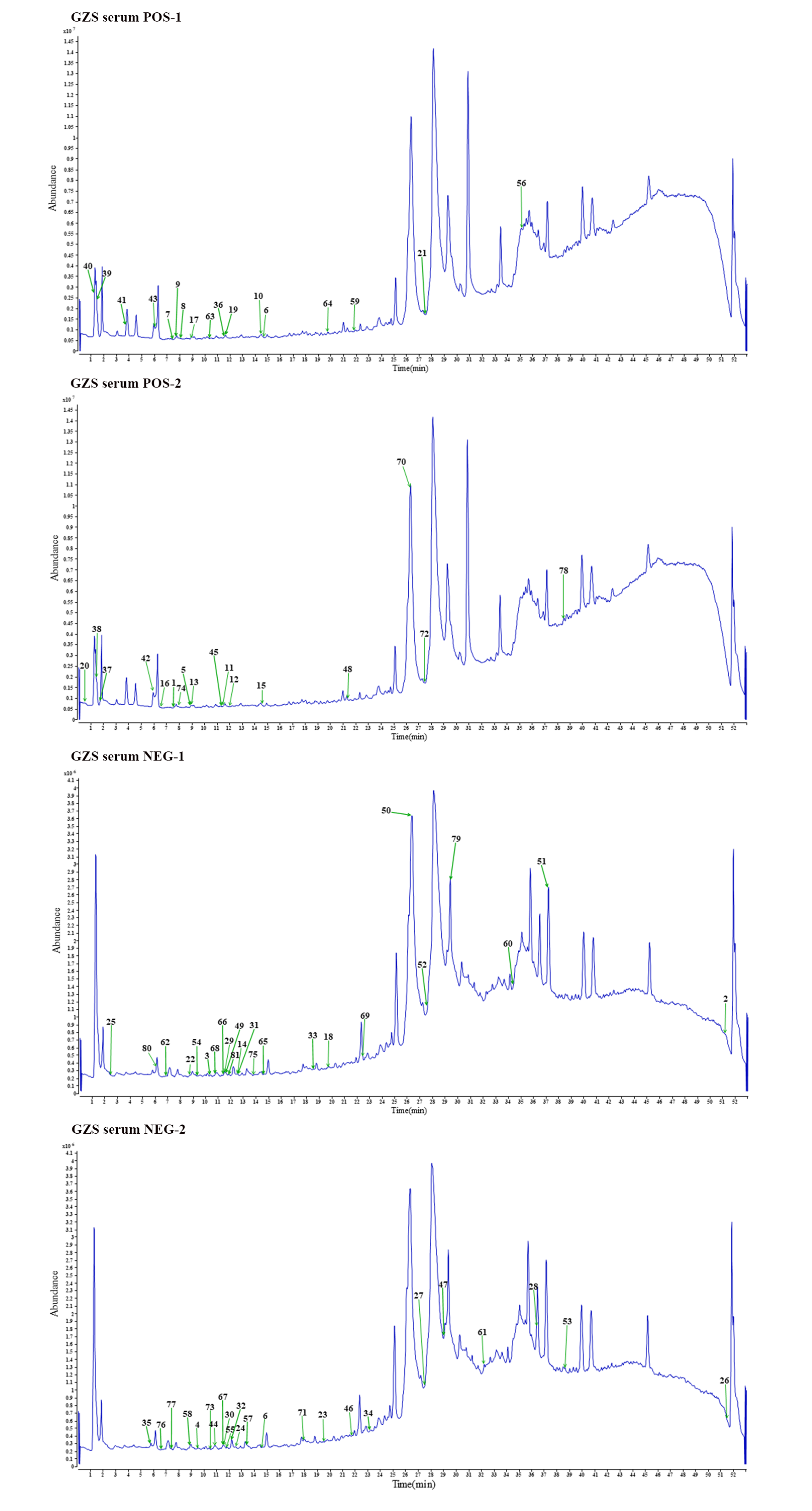


**Fig. S2** Total Ion Chromatogram of serum components


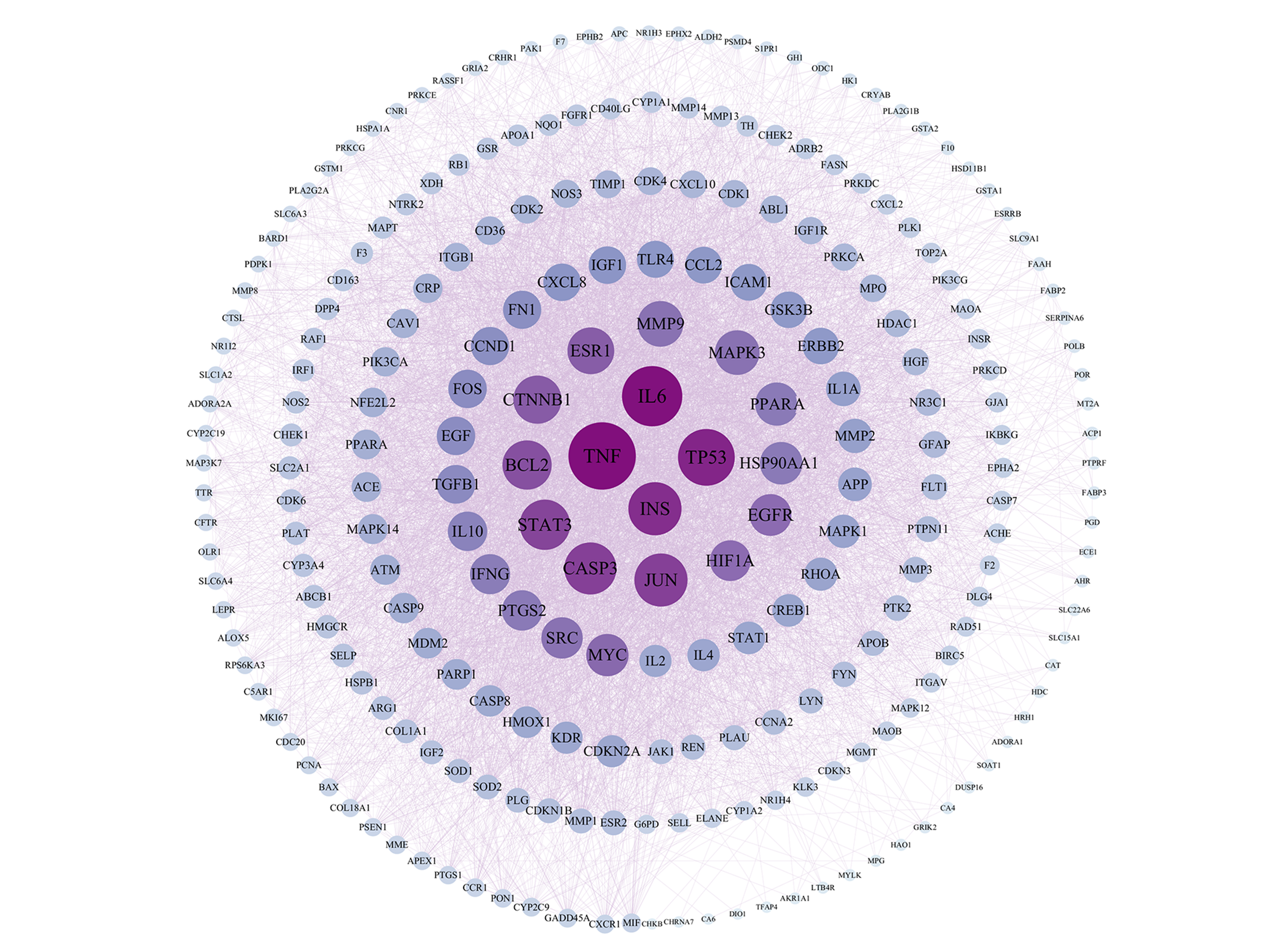


**Fig. S3** PPI


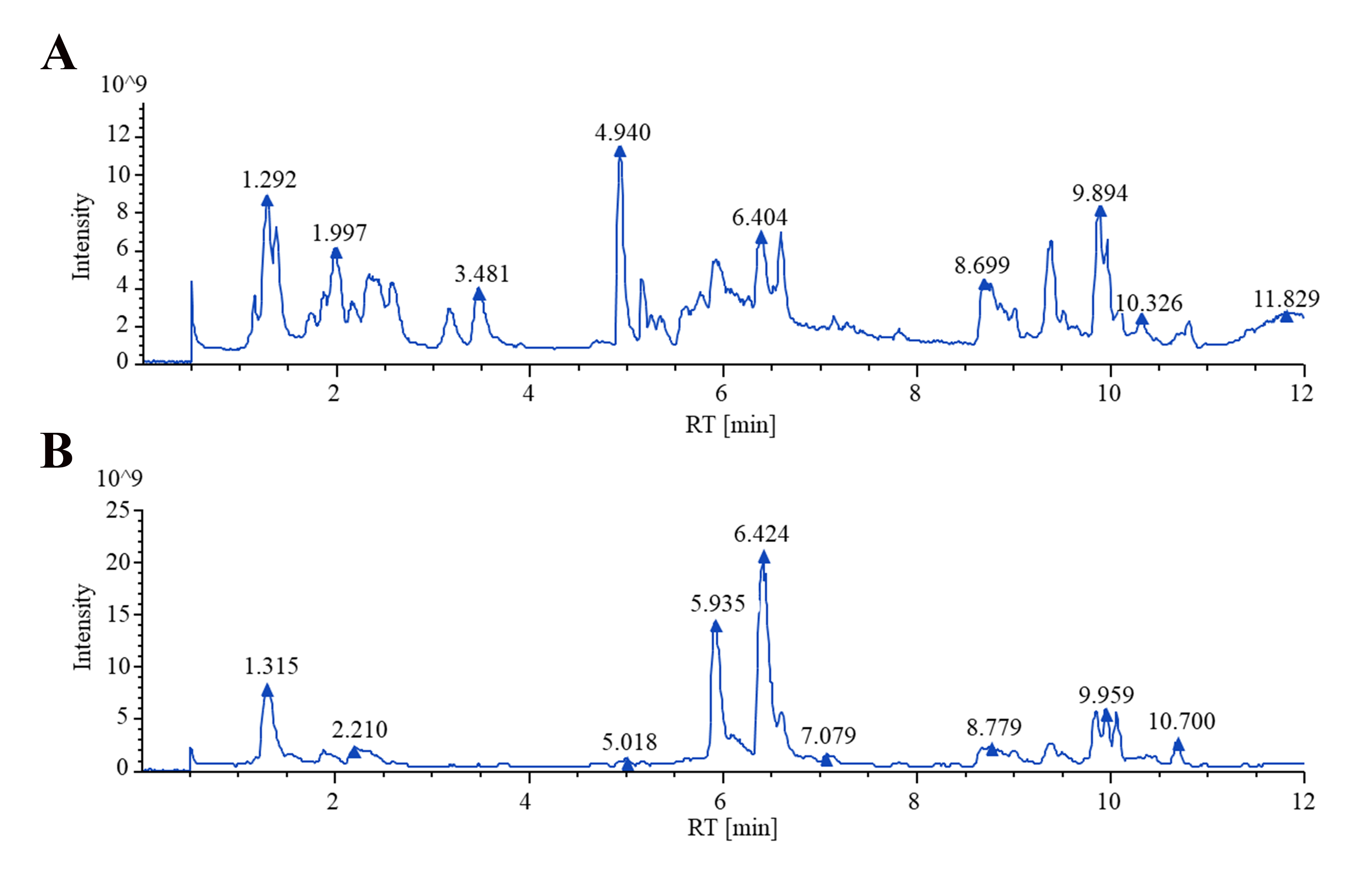


**Fig. S4** Total Ion Chromatogram of metabolites. **A**-positive mode, **B**-negative mode.


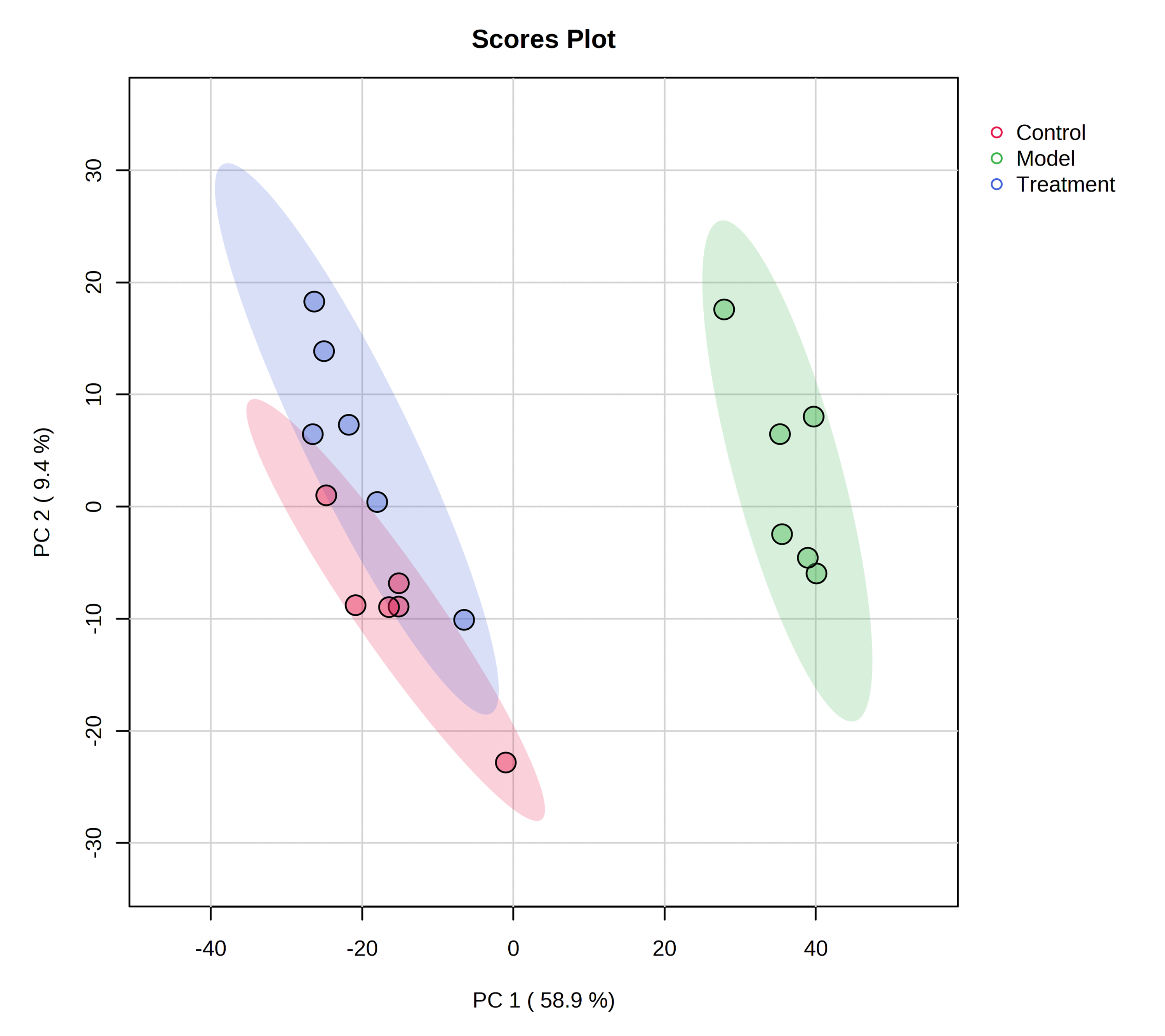


**Fig. S5** PCA score plot.


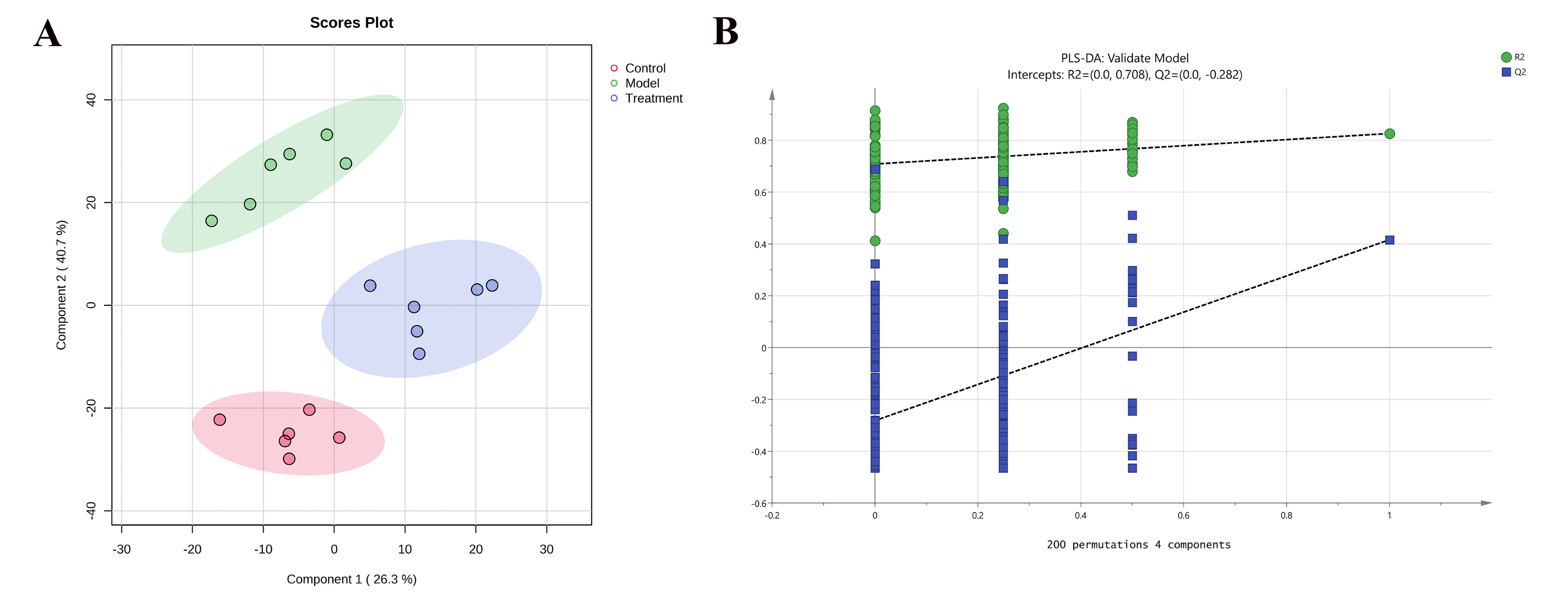


**Fig. S6** OPLS-DA model. **A**-OPLS-DA score plot, **B**-permutation test of OPLS-DA.


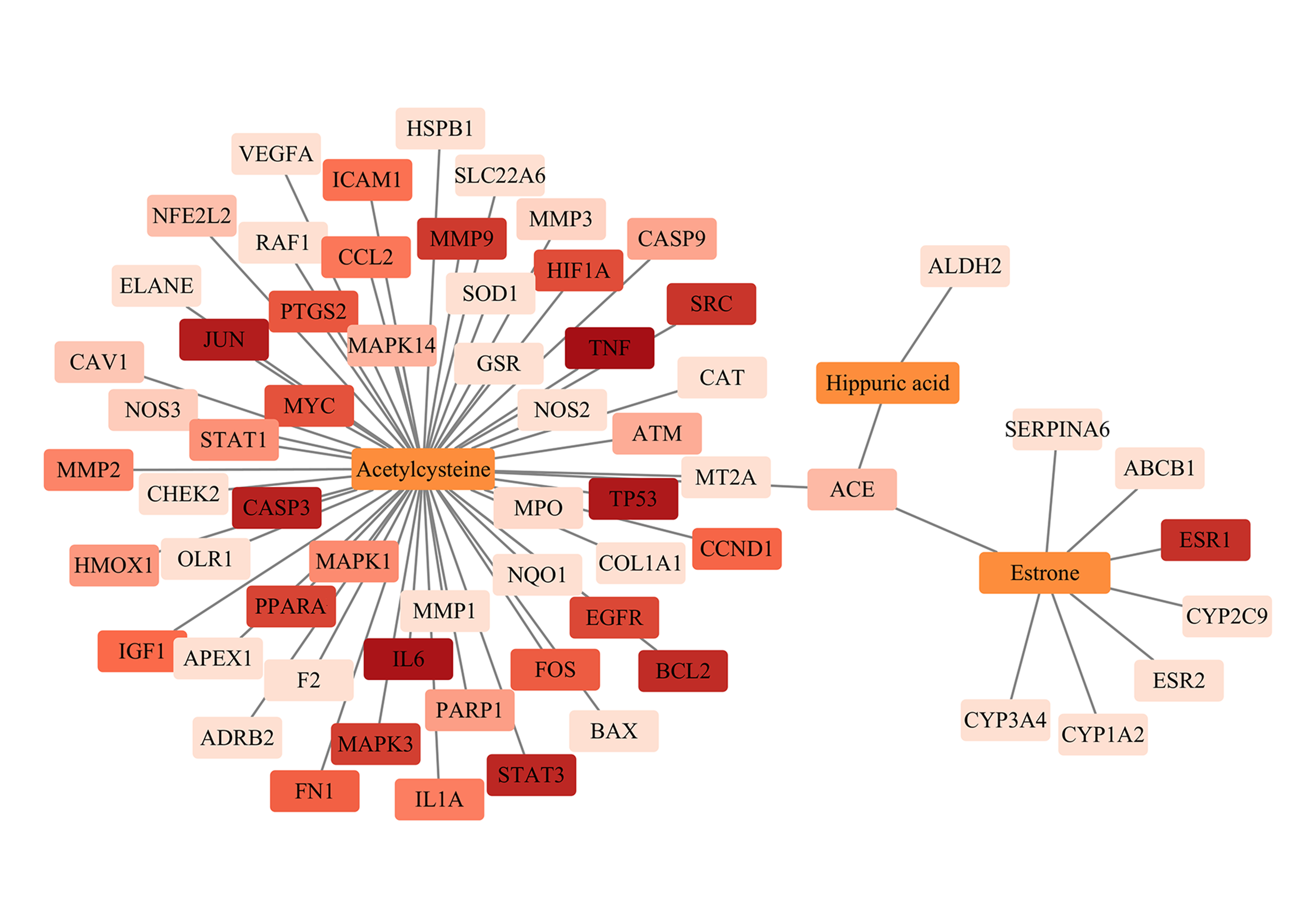


**Fig. S7** Associated analysis of genes and metabolites


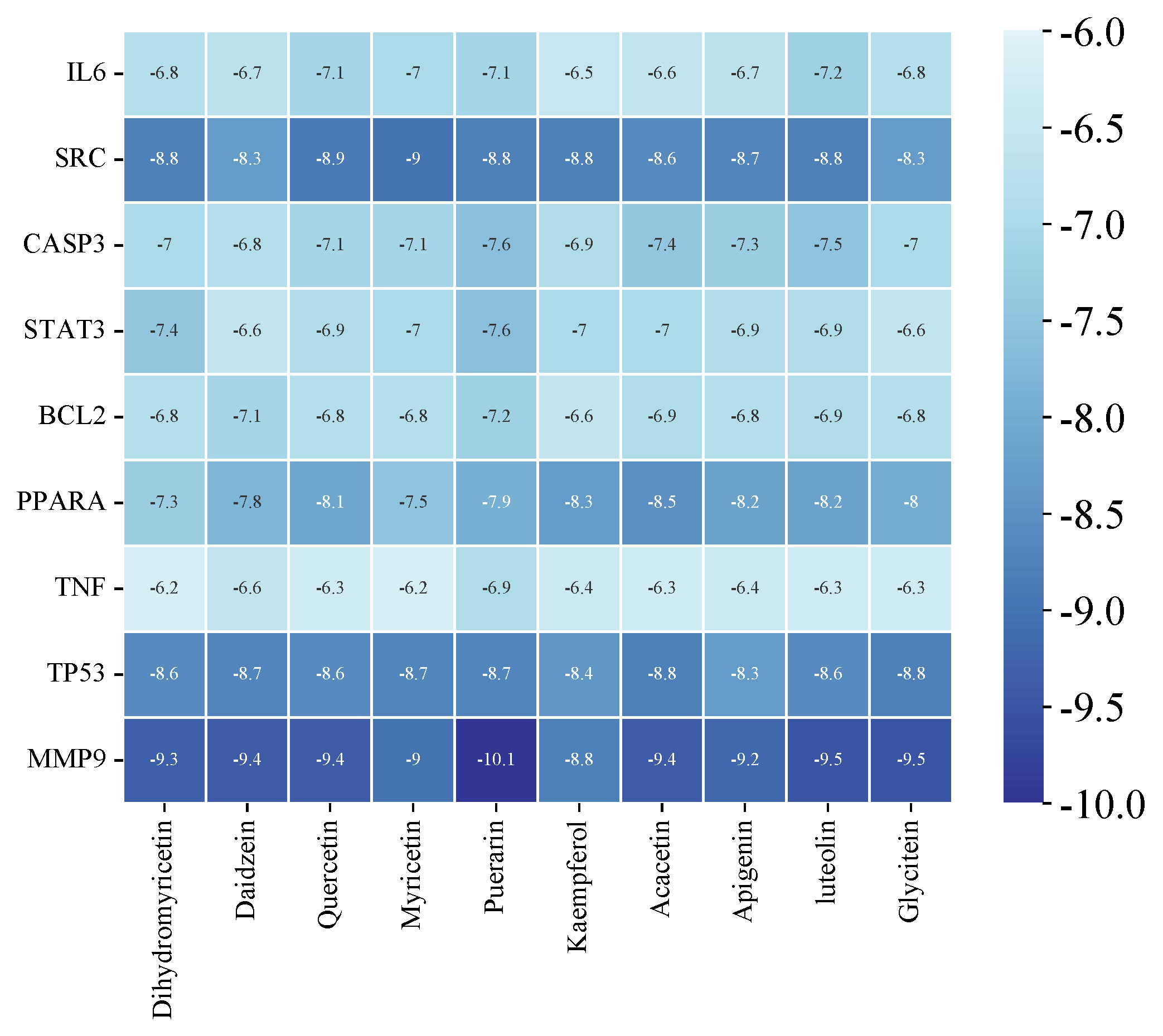


**Fig. S8** Molecular docking of 10 corresponding compounds and 9 key target proteins.


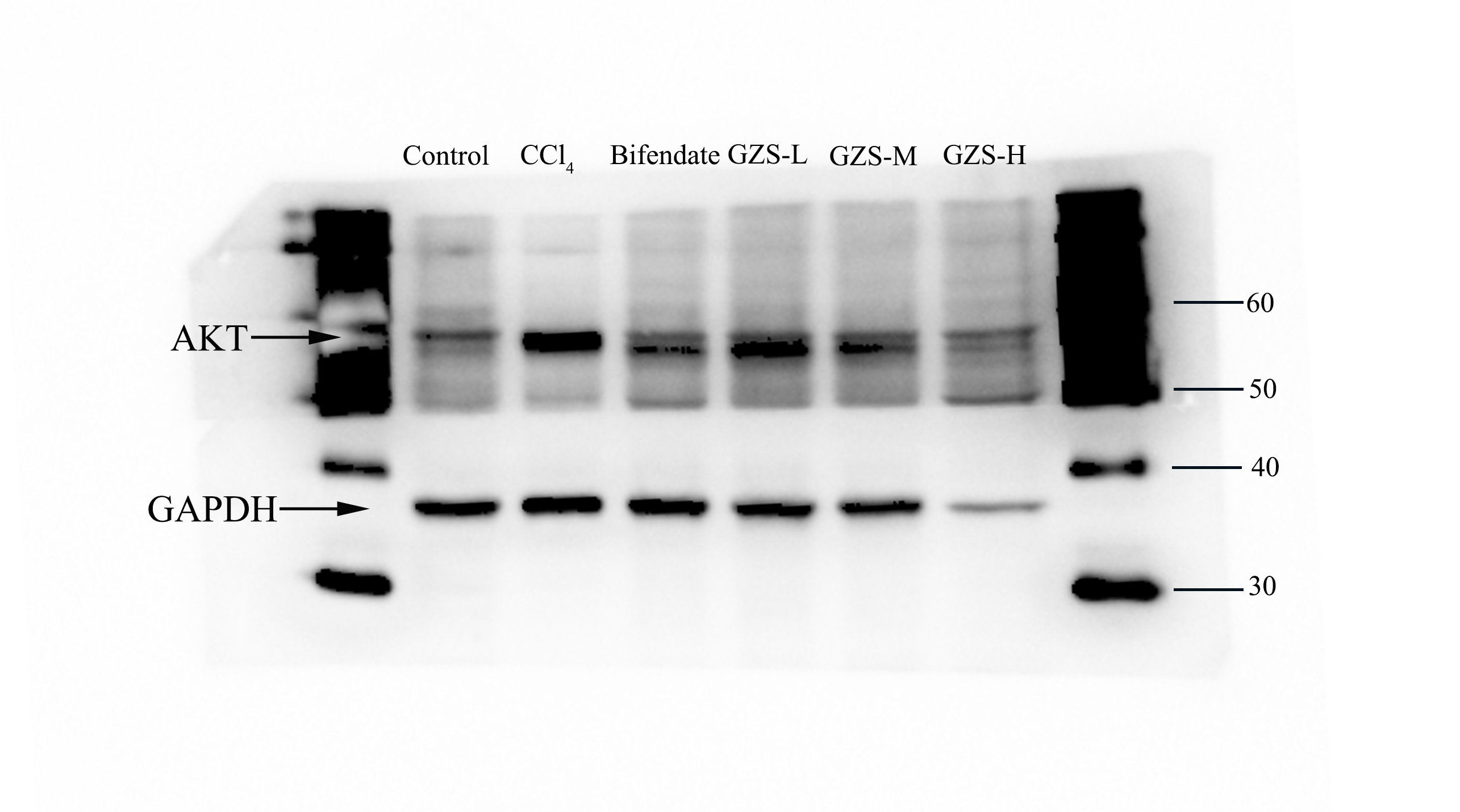


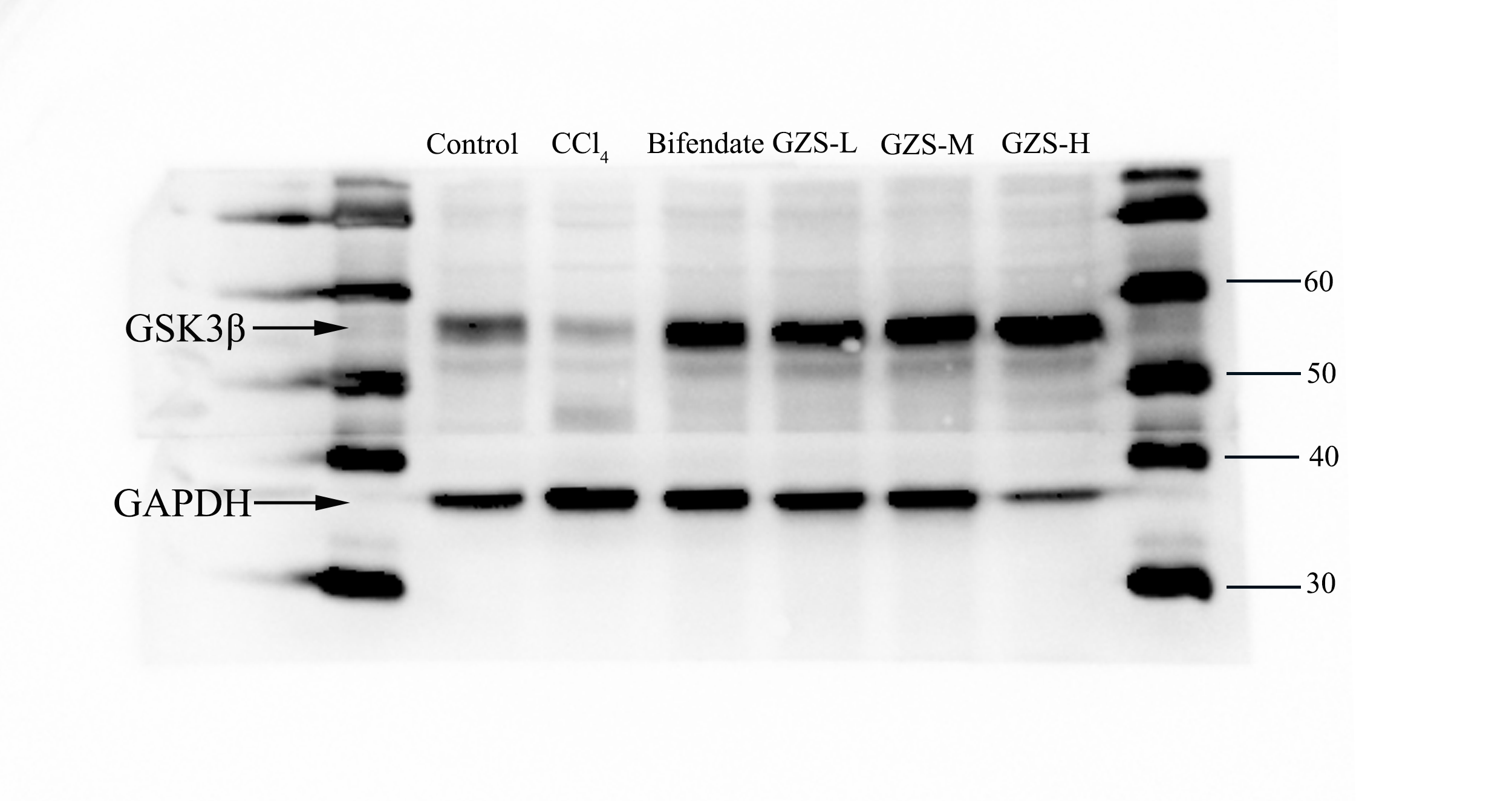


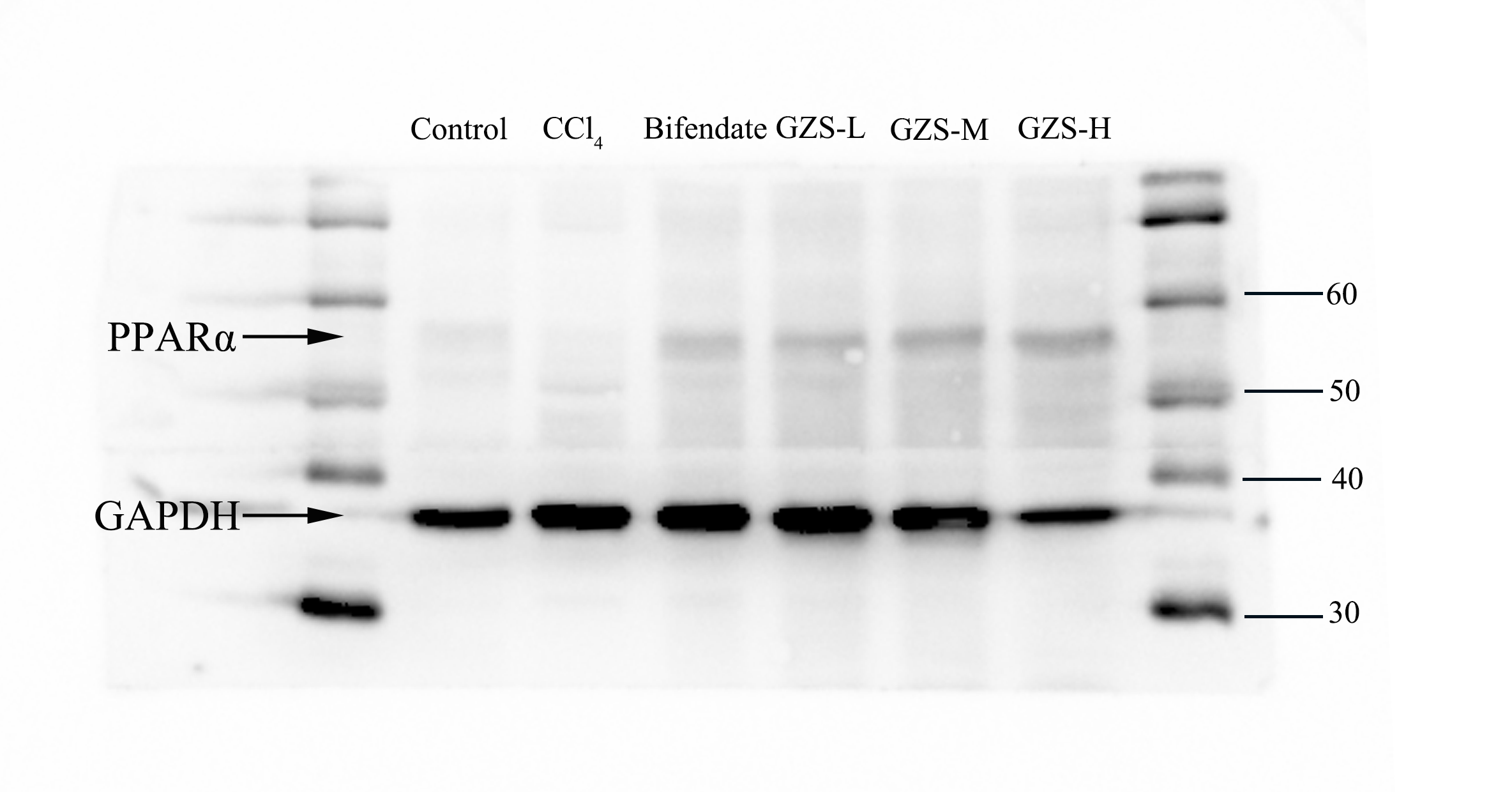


**References:**

Chen, X., Zhang, J., Li, R., Zhang, H., Sun, Y., Jiang, L., Wang, X., & Xiong, Y. (2022). Flos Puerariae-Semen Hoveniae medicinal pair extract ameliorates DSS-induced inflammatory bowel disease through regulating MAPK signaling and modulating gut microbiota composition. *Frontiers in Pharmacology*, *13*, 1034031.

Cheng, R., Sun, M., Hu, Q., Deng, Z., Zhang, B., & Li, H. (2023). Hovenia acerba Lindl. peduncles and seeds extracts ameliorate alcoholic liver injury by activating the Nrf2/HO-1 signalling pathway in LO2 cells and mice. *Food Bioscience*, *51*, 102224.

Dai, T., & Sun, G. (2022). Metabolite profiling analysis of plasma, urine, and feces of rats after oral administration of Flos Chrysanthemi Indici preparation through UHPLC-Q-Exactive-MS combined with pharmacokinetic study of markers by UHPLC-QQQ-MS/MS. *ANALYTICAL AND BIOANALYTICAL CHEMISTRY*, *414*(13), 3927-3943.

Guo, S., Duan, J. A., Tang, Y., Qian, Y., Zhao, J., Qian, D., Su, S., & Shang, E. (2011). Simultaneous qualitative and quantitative analysis of triterpenic acids, saponins and flavonoids in the leaves of two Ziziphus species by HPLC-PDA-MS/ELSD. *JOURNAL OF PHARMACEUTICAL AND BIOMEDICAL ANALYSIS*, *56*(2), 264-270.

Li, J., Xing L. J., Wang Y., Luo R. F., & Lu S. L. (2021). Study on the Analysis of Polysaccharides in Lycium ruthenicum Based on UPLC-MS/MS. *Farm Products Processing,* (12), 60-62.

Li, X., Yang, W., Chen, H., Pan, F., Liu, W., Qi, D., Yu, S., Liu, H., Chai, X., Liu, Y., Pan, Y., & Wang, G. (2023). Rapid screening and in vivo target occupancy quantitative evaluation of xanthine oxidase inhibitors based on drug-target binding kinetics research strategy: A case study of Chrysanthemum morifolium Ramat. *BIOMEDICINE & PHARMACOTHERAPY*, *161*, 114379.

Liu, T., Tian, X., Li, Z., Han, F., Ji, B., Zhao, Y., & Yu, Z. (2018). Metabolic profiling of Gegenqinlian decoction in rat plasma, urine, bile and feces after oral administration by ultra high performance liquid chromatography coupled with Fourier transform ion cyclotron resonance mass spectrometry. *JOURNAL OF CHROMATOGRAPHY B-ANALYTICAL TECHNOLOGIES IN THE BIOMEDICAL AND LIFE SCIENCES*, *1079*, 69-84.

Luo, J., Liang, L., Xie, Q., Qiu, Y., Jiang, S., Yang, Y., Zhu, L., Fu, Y., Chen, S., Wang, W., & Yuan, H. (2023). Differential analysis of phytochemistry and antioxidant activity in five citrus by-products based on chromatography, mass spectrometry, and spectrum-effect relationships. *Food Chemistry-X*, *20*, 101010.

Ouyang, H., Fan, Y., Wei, S., Chang, Y., & He, J. (2022). Study on the Chemical Profile of Chrysanthemum (Chrysanthemum morifolium) and the Evaluation of the Similarities and Differences between Different Cultivars. *CHEMISTRY & BIODIVERSITY*, *19*(8), e202200252.

Peng, H., Deng, Z., Chen, X., Sun, Y., Zhang, B., & Li, H. (2018). Major chemical constituents and antioxidant activities of different extracts from the peduncles of Hovenia acerba Lindl. *INTERNATIONAL JOURNAL OF FOOD PROPERTIES*, *21*(1), 2135-2155.

Qin, S. H., Yan, F., E, S., Xiong, P., Tang, S. N., Yu, K. Q., Zhang, M., Cheng, Y. C., & Cai, W. (2022). Comprehensive characterization of multiple components of Ziziphus jujuba Mill using UHPLC-Q-Exactive Orbitrap Mass Spectrometers. *Food Science & Nutrition*, *10*(12), 4270-4295.

Shi, Z., Li, Z., Zhang, S., Fu, H., & Zhang, H. (2015). Subzero-Temperature Liquid-Liquid Extraction Coupled with UPLC-MS-MS for the Simultaneous Determination of 12 Bioactive Components in Traditional Chinese Medicine Gegen-Qinlian Decoction. *JOURNAL OF CHROMATOGRAPHIC SCIENCE*, *53*(8), 1407-1413.

Sun, X., Deng, H., Shan, B., Shan, Y., Huang, J., Feng, X., Tang, X., Ge, Y., Liao, P., & Yang, Q. (2023). Flavonoids contribute most to discriminating aged Guang Chenpi (Citrus reticulata 'Chachi') by spectrum-effect relationship analysis between LC-Q-Orbitrap/MS fingerprint and ameliorating spleen deficiency activity. *Food Science & Nutrition*, *11*(11), 7039-7060.

Wang, H. P., Lin, Z. Z., Wang, H., Yang, X., & Niu, N. (2024). Comprehensive identifying flavonoids in Citri Reticulatae Pericarpium using a novel strategy based on precursor ions locked and targeted MS/MS analysis. *Scientific Reports*, *14*(1), 9679.

Xiao, X., Ren, W., Zhang, N., Bing, T., Liu, X., Zhao, Z., & Shangguan, D. (2019). Comparative Study of the Chemical Constituents and Bioactivities of the Extracts from Fruits, Leaves and Root Barks of Lycium barbarum. *MOLECULES*, *24*(8).

Zhang, G., Chen, S., Zhou, W., Meng, J., Deng, K., Zhou, H., Hu, N., & Suo, Y. (2018). Rapid qualitative and quantitative analyses of eighteen phenolic compounds from Lycium ruthenicum Murray by UPLC-Q-Orbitrap MS and their antioxidant activity. *FOOD CHEMISTRY*, *269*, 150-156.

Zhang, Z. T., Guo, N., Zhuang, G. D., Deng, S. M., He, W. J., Chen, Z. Q., Xu, Y. H., Tang, D., & Wang, S. M. (2021). Metabolic Profiling of Carbonyl Compounds for Unveiling Protective Mechanisms of Pueraria lobata against Diabetic Nephropathy by UPLC-Q-Orbitrap HRMS/MS Analysis. *JOURNAL OF AGRICULTURAL AND FOOD CHEMISTRY*, *69*(37), 10943-10951.
